# Phosphorylation and arginine methylation mark histone H2A prior to deposition during *Xenopus laevis* development

**DOI:** 10.1186/1756-8935-7-22

**Published:** 2014-09-06

**Authors:** Wei-Lin Wang, Lissa C Anderson, Joshua J Nicklay, Hongshan Chen, Matthew J Gamble, Jeffrey Shabanowitz, Donald F Hunt, David Shechter

**Affiliations:** 1 Department of Biochemistry, Albert Einstein College of Medicine of Yeshiva University, Bronx, NY 10461, USA; 2 Department of Molecular Pharmacology, Albert Einstein College of Medicine of Yeshiva University, Bronx, NY 10461, USA; 3 Department of Chemistry, Health Sciences Center, University of Virginia, Charlottesville, VA 22904, USA; 4 Department of Pathology, Health Sciences Center, University of Virginia, Charlottesville, VA 22904, USA

**Keywords:** Histones, H2A, *Xenopus laevis*, Histone arginine methylation, Histone phosphorylation, Histone deposition, Early development

## Abstract

**Background:**

Stored, soluble histones in eggs are essential for early development, in particular during the maternally controlled early cell cycles in the absence of transcription. Histone post-translational modifications (PTMs) direct and regulate chromatin-templated transactions, so understanding the nature and function of pre-deposition maternal histones is essential to deciphering mechanisms of regulation of development, chromatin assembly, and transcription. Little is known about histone H2A pre-deposition modifications nor known about the transitions that occur upon the onset of zygotic control of the cell cycle and transcription at the mid-blastula transition (MBT).

**Results:**

We isolated histones from staged *Xenopus laevis* oocytes, eggs, embryos, and assembled pronuclei to identify changes in histone H2A modifications prior to deposition and in chromatin. Soluble and chromatin-bound histones from eggs and embryos demonstrated distinct patterns of maternal and zygotic H2A PTMs, with significant pre-deposition quantities of S1ph and R3me1, and R3me2s. We observed the first functional distinction between H2A and H4 S1 phosphorylation, as we showed that H2A and H2A.X-F (also known as H2A.X.3) serine 1 (S1) is phosphorylated concomitant with germinal vesicle breakdown (GVBD) while H4 serine 1 phosphorylation occurs post-MBT. In egg extract H2A/H4 S1 phosphorylation is independent of the cell cycle, chromatin assembly, and DNA replication. H2AS1ph is highly enriched on blastula chromatin during repression of zygotic gene expression while H4S1ph is correlated with the beginning of maternal gene expression and the lengthening of the cell cycle, consistent with distinct biological roles for H2A and H4 S1 phosphorylation. We isolated soluble H2A and H2A.X-F from the egg and chromatin-bound in pronuclei and analyzed them by mass spectrometry analysis to quantitatively determine abundances of S1ph and R3 methylation. We show that H2A and H4 S1ph, R3me1 and R3me2s are enriched on nucleosomes containing both active and repressive histone PTMs in human A549 cells and *Xenopus* embryos.

**Conclusions:**

Significantly, we demonstrated that H2A phosphorylation and H4 arginine methylation form a new class of *bona fide* pre-deposition modifications in the vertebrate embryo. We show that S1ph and R3me containing chromatin domains are not correlated with H3 regulatory PTMs, suggesting a unique role for phosphorylation and arginine methylation.

## Background

Histone post-translational modifications (PTMs) serve important functions for regulation of gene expression, DNA repair, and chromatin structure. PTMs alter the charge of chromatin and/or serve as binding or docking sites for effector proteins. Histone PTMs specifically promote chromatin condensation for mitosis, specify DNA replication origins, and regulate recruitment of effectors to promote or repress gene expression [1-4].

Modifications of histone H2A and its variants are less well characterized than those on H3 and H4. Both H2A and H4 share the same five N-terminal amino acids, SGRGK, containing three modifiable residues. H2A/H4 serine 1 phosphorylation (S1ph) is highly enriched in mitotic chromatin in worms, flies, and mammals [5]. H2A/H4S1ph also exists in chromatin during interphase and S phase and is hypothesized to participate in alteration of chromatin structure for DNA repair, recombination, or gene expression. This modification can be detected with an antibody that recognizes the epitope in both H2A and H4 [5,6].

The biological function of H2A S1ph during embryogenesis is unknown. H2A/H4 S1ph may have redundant functions with H1 and H3 phosphorylation during mitosis [5] as H2A S1ph and H3 S10ph both can be phosphorylated by MSK1 [7,8]. Histone H4 serine 1 phosphorylation is highly enriched during sporulation in yeast. An H4 S1A mutant has reduced sporulation efficiency and generates aberrant spores. Analysis from budding yeast, fly, and mouse studies indicates H4S1ph may be involved in gamete-associated packaging of chromatin [9]. H4S1ph has a similar global enrichment pattern as H4K12 acetylation during sporulation in budding yeast. Those two marks were enriched in transcription-start sites compared with the transcribed downstream regions. Further analysis revealed that H4S1ph is necessary for re-repression of sporulation-specific genes after their peak expression [10].

Histone H2A arginine 3 methylation (R3me1, R3me2s, and R3me2a) is catalyzed by PRMT1 and PRMT5 *in vitro*[11-13], but few studies had neither shown its existence *in vivo* nor tested if this PTM occurs pre- or post-deposition. Histone H2A lysine 5 acetylation (K5ac) is also poorly documented. None of these H2A modifications have any presently known biological function.

Histone PTMs have been extensively studied in cultured cells. Only a few studies have probed the nature of these PTMs during vertebrate development and are primarily focused on H3 and H4 [14]. Our work previously demonstrated the enrichment of a wide range of histone PTMs across discrete developmental stages in living frog embryos and in adult cultured cells, demonstrating the remarkable discrimination of cell type by the histone code. Furthermore, we also probed the developmental histone H3 and H4 PTM transitions by examining stored oocyte and egg histones, sperm histones, and pronuclear (early-embryo equivalent) histones [6,15].

Early *Xenopus* embryonic development is tightly regulated to prevent pre-mature maternal transcripts post-germinal vesicle breakdown (GVBD) and to activate maternal gene expression in a timely manner, post mid-blastula transition (MBT). There are two major H2As in gametes and early embryos of *Xenopus laevis*, H2A and H2A.X-F (also now annotated as H2A.X.3 [16]). H2A.X-F is distinct from H2A.X as the C-terminal consensus sequence is ‘SQEF’ instead of ‘SQEY’. It is present in late-stage oocytes, in eggs, and in early embryos in large quantity (>50% of the H2A). It is only maternally expressed and is therefore diluted in chromatin by expression of canonical H2A during developmental cell division. It is not observed after the late tailbud stage. We previously showed that the phosphorylation on the C-terminal serine of H2A.X-F (SphQEF) occurs in eggs and is independent of DNA damage signals and linked to normal embryonic development [15].

*Xenopus laevis* is a significant model organism for early development and is uniquely suited for studying pre- and post-deposition histones due to the large store of maternal chromatin components in the egg. Here, we isolated chromatin from developmentally staged *Xenopus laevis* oocytes, eggs, embryos, and pronuclei assembled in cell-free egg extract. We developed a new technique to separate free soluble, chaperone-bound histones from those in chromatin in *Xenopus* embryos. We probed these samples with specific antibodies and with high-resolution mass spectrometry. We demonstrate that unique H2A and H4 modification signatures occur during oogenesis prior to incorporation, including S1ph and R3me1/2 s, and are enriched in deposited histones during the early developmental transcriptional quiescence.

## Results

### H2A post-translational modifications in chromatin assembly in cell free egg extract

**Figure 1:**
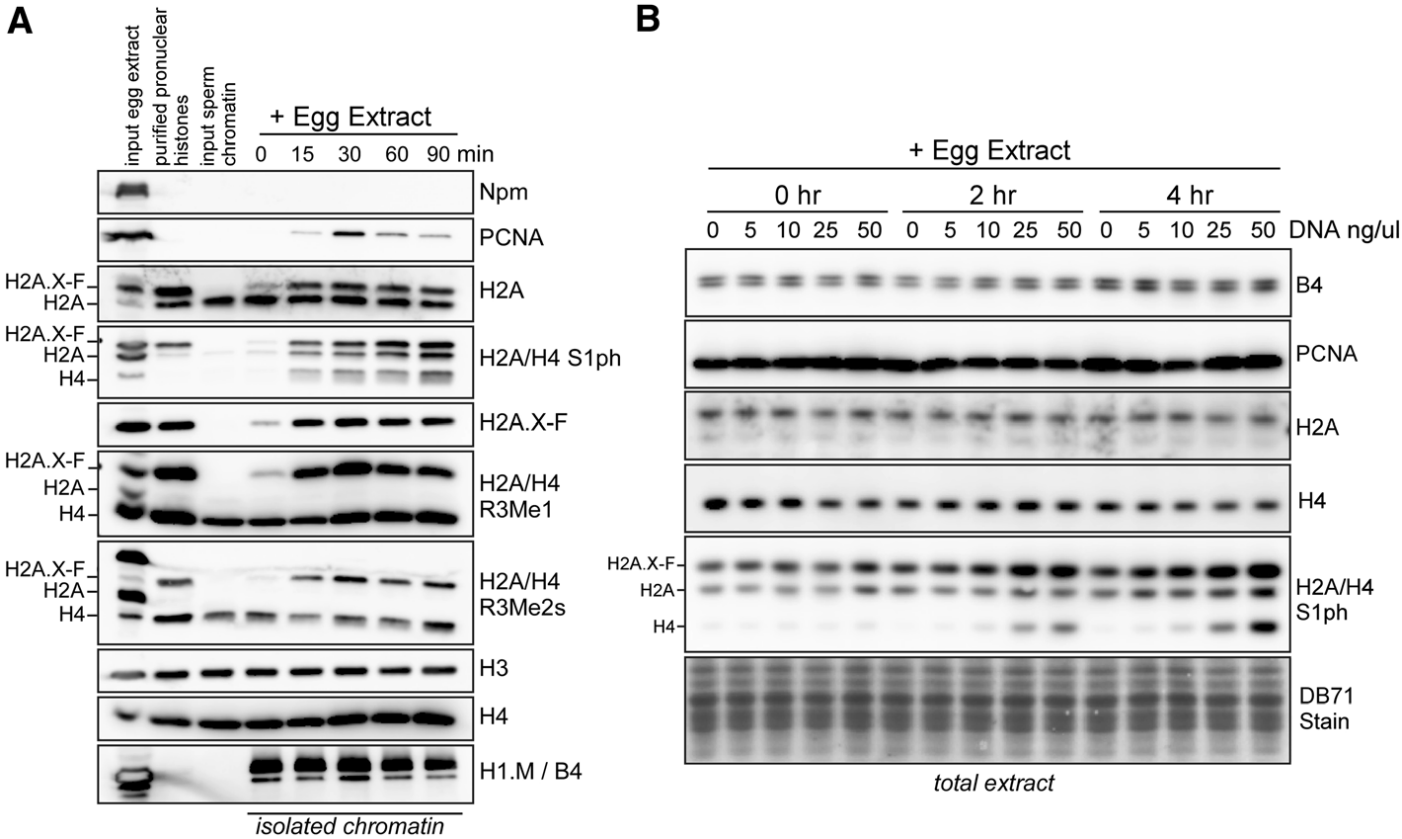
**H2A and H2A.X-F S1 phosphorylation and R3 methylation are enriched on pronuclei, while H4 S1 phosphorylation is DNA concentration dependent. (A)** Pronuclei were assembled in egg extract and chromatin was isolated through a sucrose cushion at 0, 15, 30, 60, and 90 min post incubation. Isolated chromatin proteins were immunoblotted as shown (right five lanes). Input egg extract, purified pronuclear histones, and sperm histones were also immunoblotted (left three lanes). **(B)** Egg extract was incubated with increasing concentrations of plasmid DNA (0, 5, 10, 25, and 50 ng/μL) for 0, 2, or 4 h. Samples of the total reaction were immunoblotted as shown.

We initially investigated the changes in H2A PTMs during pronuclear assembly in egg extract. Note that H2A, H2A.X-F, and H4 both contain the same five amino acid N-terminus (Additional file 1: Figure S1). A pronuclei formation assay was performed to compare stored histones in extract and chromatin bound histones by immunoblot (Figure 1A). In the first three lanes we blotted the total input egg extract, heparin purified egg histones [6,17], and sucrose cushion isolated sperm histones. Assembled pronuclei were isolated from egg extract through a sucrose cushion at 0, 15, 30, 60, and 90 min post-sperm addition (Figure 1A, right five lanes). Equivalent volumes of extract or isolated chromatin were run on the gel, with H2A, H3, and H4 immunoblots as loading controls. During the course of pronuclear assembly we observed accumulation of H2A.X-F arginine methylation on chromatin. We also showed that H2A.X-F S1ph was preferentially loaded compared to H2A S1ph in chromatin during assembly. We also observed rapid deposition of linker histone H1.M (also known as B4).

To determine if H2A/H2A.X-F and H4 S1 phosphorylation was DNA concentration or chromatin assembly dependent, we incubated cell-free egg extract with 0, 5, 10, 25, 50 ng/μL plasmid DNA for 0, 2, or 4 h and ran equivalent volumes of each reaction on a gel (Figure 1B). We blotted H1.M, PCNA, H2A, and H4 as controls and did not observe any DNA incubation-dependent alteration in abundance. Loading levels were controlled by the DB71 membrane stain (bottom panel). Strikingly, when we probed S1 phosphorylation we observed constant H2A S1ph, and increasing H2A.X-F and H4 S1ph in a DNA concentration-dependent fashion. The S1ph antibody equally recognized H2AS1ph and H4S1ph (Additional file 1: Figure S2). We observed an absolute requirement for >25 ng/μL DNA concentration for H4 S1ph appearance, consistent with the expected DNA concentration at the MBT and as previously shown to be saturating conditions for chromatin assembly in egg extract [18]. We conclude that H2A S1ph is generally enriched on chromatin and occurs independent of chromatin assembly, while H4 S1ph has an independent pathway, possibly connected to the nuclear-to-cytoplasmic ratio.

### The incorporation of H2A Ser 1 phosphorylation is independent of DNA replication and cell cycle transitions

**Figure 2:**
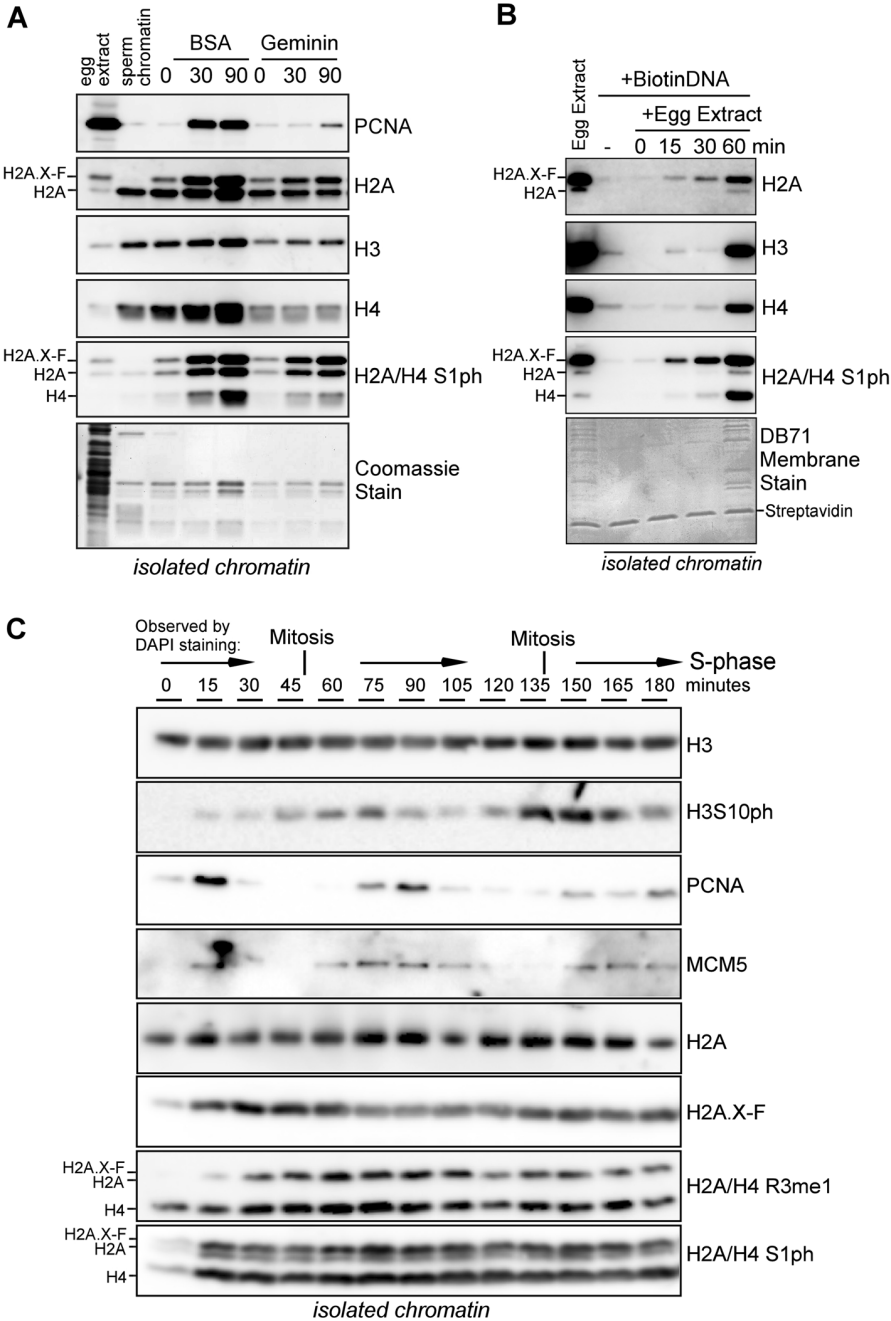
**H2A and H2A.X-F S1 phosphorylation is independent of DNA replication and the cell cycle. (A)** Sperm chromatin was incubated in egg extract with the addition of BSA (control) or the addition of 150 ng of Geminin to inhibit DNA replication. The reactions were flash-frozen at 0, 30, or 90 min and chromatin was isolated through a sucrose cushion and immunoblotted as shown. The migration positions of H2A.X-F, H2A, and H4 are indicated. **(B)** Double-stranded DNA bound to streptavidin beads was incubated in egg extract for 0, 15, 30, or 60 min, then isolated and washed. Precipitated protein was immunoblotted for H2A, H3, H4, and S1ph as indicated. **(C)** Sperm chromatin was incubated in cycling egg extract and aliquots were flash-frozen every 15 min, from 0 to 180 min. Chromatin was isolated through a sucrose cushion and immunoblotted as shown. Interphase and mitosis were observed by DAPI-stained chromatin and noted at the top of the panel.

To test if H2A S1 phosphorylation occurs during DNA replication, we probed histones isolated from pronuclei either in S-phase or incubated with Geminin protein, an inhibitor of S-phase progression [19]. As shown in Figure 2A, we isolated pronuclei at 0, 30, and 90 min post sperm chromatin addition in the presence of BSA (control) or 1.875 μg/mL Geminin. Each lane contains equivalent volume of chromatin protein isolated through a sucrose cushion. Input egg extract and input sperm chromatin were run on the gel as controls. First, we probed for PCNA presence on the chromatin as an indication of ongoing DNA replication. PCNA abundance was well correlated with ongoing DNA replication in the BSA control samples and was reduced or not present in the Geminin treated samples, confirming the block to DNA replication. Additionally, H2A, H3, and H4 total abundances increased concomitant with DNA replication, as expected for histones bound to the duplicated DNA.

To further demonstrate the independence of S1ph from DNA replication, we incubated double-stranded biotinylated DNA in egg extract and isolated the assembled chromatin on streptavidin resin at 0, 15, 30, and 60 min after addition (Figure 2B). Egg extract does not replicate small DNAs, but does assemble chromatin as shown by the capture of histones. We also observed S1ph on the isolated chromatin, concomitant with assembly. Therefore we concluded that deposition of H2A S1ph is independent of DNA replication in *Xenopus* egg extract.

To determine if S1 phosphorylation is dependent on other cell cycle transitions, including progression through mitosis, we prepared cycling cell-free egg extract and incubated sperm chromatin at 3,000/μL (Figure 2C). From 0 to 180 min we flash-froze 150 μL aliquots of extract and simultaneously observed progression through the cell cycle via DAPI staining and microscopy (observations indicated at the top of the panel in Figure 2B). We then isolated chromatin from the flash-frozen samples through a sucrose cushion and immunoblotted as indicated. This is the first demonstration of the isolation of chromatin from cycling extract. To confirm cell cycle transitions we probed isolated chromatin with known markers. H3 S10 phosphorylation abundance peaked during the observed mitosis, consistent with our expectation [20-22]. PCNA, a critical component of the DNA polymerase complex, and MCM5, a component of the replicative helicase, peaked in abundance on chromatin during observed interphase. Histones H3, H2A, and H2A.X-F did not exhibit cyclical changes in abundance on chromatin, as expected. We also probed R3me1 and S1ph and did not observe any cell-cycle-dependent changes in chromatin abundance (Figure 2C, bottom panels).

From these experiments we conclude that the incorporation of H2A/H2A.X-F S1ph is independent of DNA replication and that H2A/H4 R3me and S1ph are independent of the cell cycle transitions during mitosis and DNA replication.

### Developmental changes in H2A PTMs

Our initial observations led us to question if living embryos have the modifications that we observed in purified chromatin from pronuclei assembled in egg extract. The pronuclear assembly assay is an imperfect mimic of actual developmental transitions, as existing PTMs on sperm histones can bias our observations as these residual histones make up a larger proportion of the total population. Additionally, pronuclear assembly may mimic conditions both prior to and after the mid-blastula transition as the nuclear-to-cytoplasmic ratio is an important component of those molecular events [23]. Therefore, analysis of histone H2A and its variants in oocytes, eggs, and embryos will give unbiased results to probe their natural abundances and inform future studies for their biological function.

**Figure 3:**
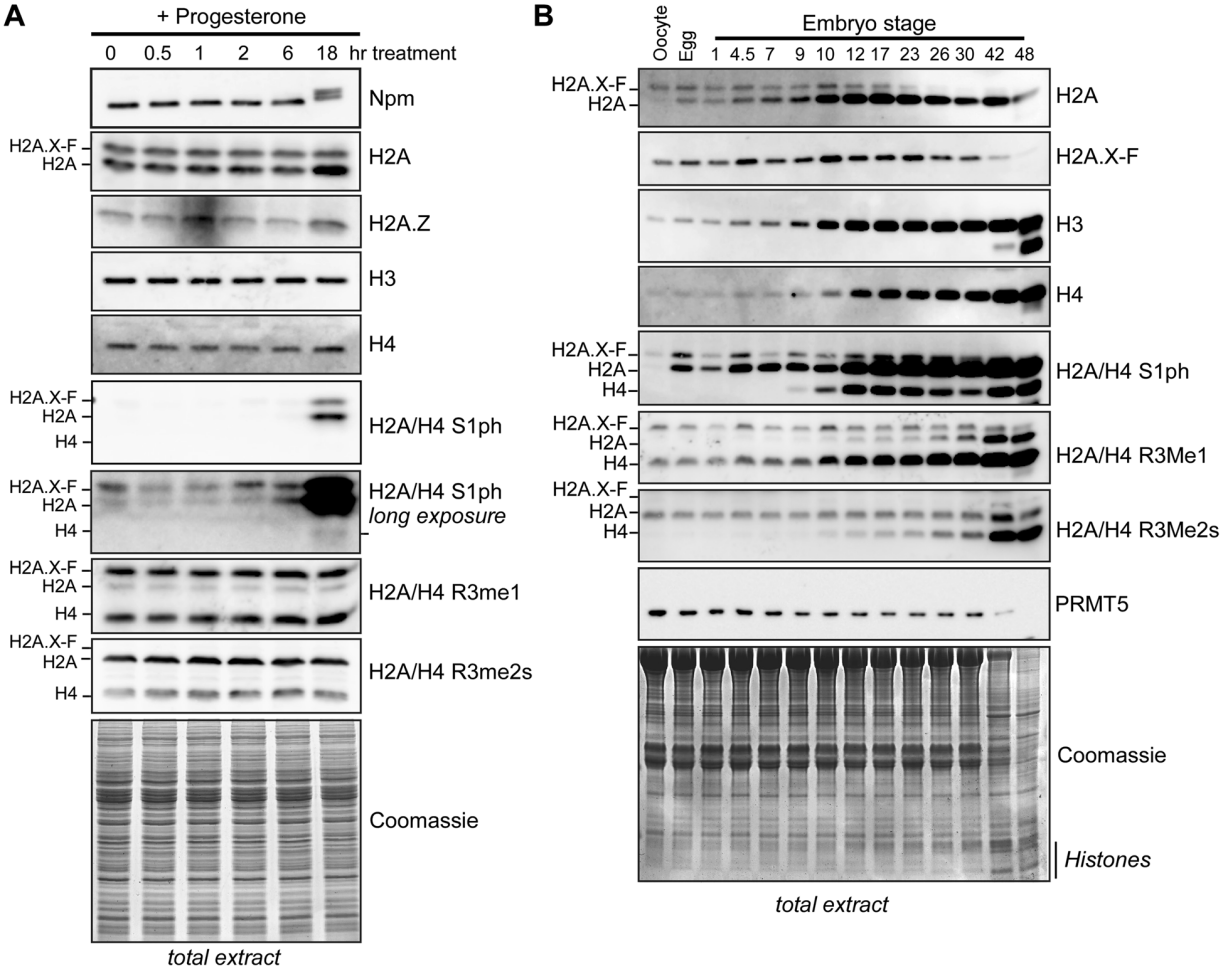
**H2A and H2A.X-F are dynamically modified during oocyte maturation and early development. (A)** Stage VI oocytes were treated with 15 μM progesterone and samples from 0, 0.5, 1, 2, 6, and 18 h post-treatment were collected, frozen, and lysed for immunoblot analysis as shown. Total protein is shown in the Coomassie stained gel at the bottom. The migration positions of H2A.X-F, H2A, and H4 are indicated. **(B)** Pooled oocytes, eggs, and fertilized embryos through stage 48 were collected, frozen, and lysed. Total protein samples were immunoblotted as shown. Total protein is shown in the Coomassie stained gel at the bottom. The migration positions of H2A.X-F, H2A, and H4 are indicated.

We first investigated modifications to histone H2A and H2A.X-F upon maturation of oocytes to eggs through GVBD. To promote GVBD, we isolated stage VI oocytes from mature female frogs and treated them with 15 μM progesterone. Five maturing oocytes were collected at 0, 0.5, 1, 2, 6, and 18 h post-treatment, and flash-frozen. The samples were lysed in 1X SDS-PAGE loading buffer and boiled, followed by immunoblot analysis (Figure 3A). We observed hyperphosphorylation of Nucleoplasmin upon GVBD confirming that progesterone treatment was effective (JN, DFH, DS, unpublished observations). All core histones remained roughly constant in abundance. We probed H2A/H4 R3me1 and R3me2s and did not observe a GVBD-dependent change in abundance. We note that Arg 3 monomethylation was enriched on H2A.X-F, while Arg 3 symmetric dimethylation was enriched on canonical H2A. Strikingly, H2A and H2A.X-F Ser 1 phosphorylation (S1ph), but not H4 Ser 1 phosphorylation, dramatically increased in abundance 18 h post treatment, concomitant with the morphological changes in the egg post GVBD. This is the first evidence showing a developmental regulation of H2A S1ph in a vertebrate. We also did not observe enrichment of H2A/H4S1ph in chromatin assembled in oocyte extract (Additional file 1: Figure S3).

H2A Ser1 phosphorylation was previously observed in mammalian cells throughout the cell cycle [5] and as we observed in *Xenopus* pronuclei, along with H2A R3 methylation [6]. We therefore probed further changes in H2A PTMs post fertilization. Oocytes, eggs, and staged embryos through the tadpole stage 48 were pooled, flash-frozen, and lysed in 1X SDS-PAGE loading buffer and boiled. Equivalent embryo fraction volumes were run on an SDS-PAGE gel, allowing us to observe the exponential increase in total histone content (Figure 3B). Note the dramatic increase in core histones H2A, H3, H4, and in the Coomassie stained gel post mid-blastula transition (MBT, observed roughly at stage 9), when the zygotic genome activation occurs. We observed the expected change from equivalent abundance of H2A and H2A.X-F to mostly H2A post MBT [15]. Interestingly, we saw dramatic changes in H2A, H2A.X-F, and H4 R3me1 and R3me2s abundances in these total extract fractions, while the abundance of the major symmetric-dimethyl arginine methyltransferase PRMT5 decreased late in development. Most strikingly, we observed hyperphosphorylation of H2A and H2A.X-F1 Ser 1 in eggs and only observed H4 Ser 1 phosphorylation post MBT.

### The deposition of soluble stores of H2A/H4 S1ph and R3me are tightly regulated throughout development

**Figure 4:**
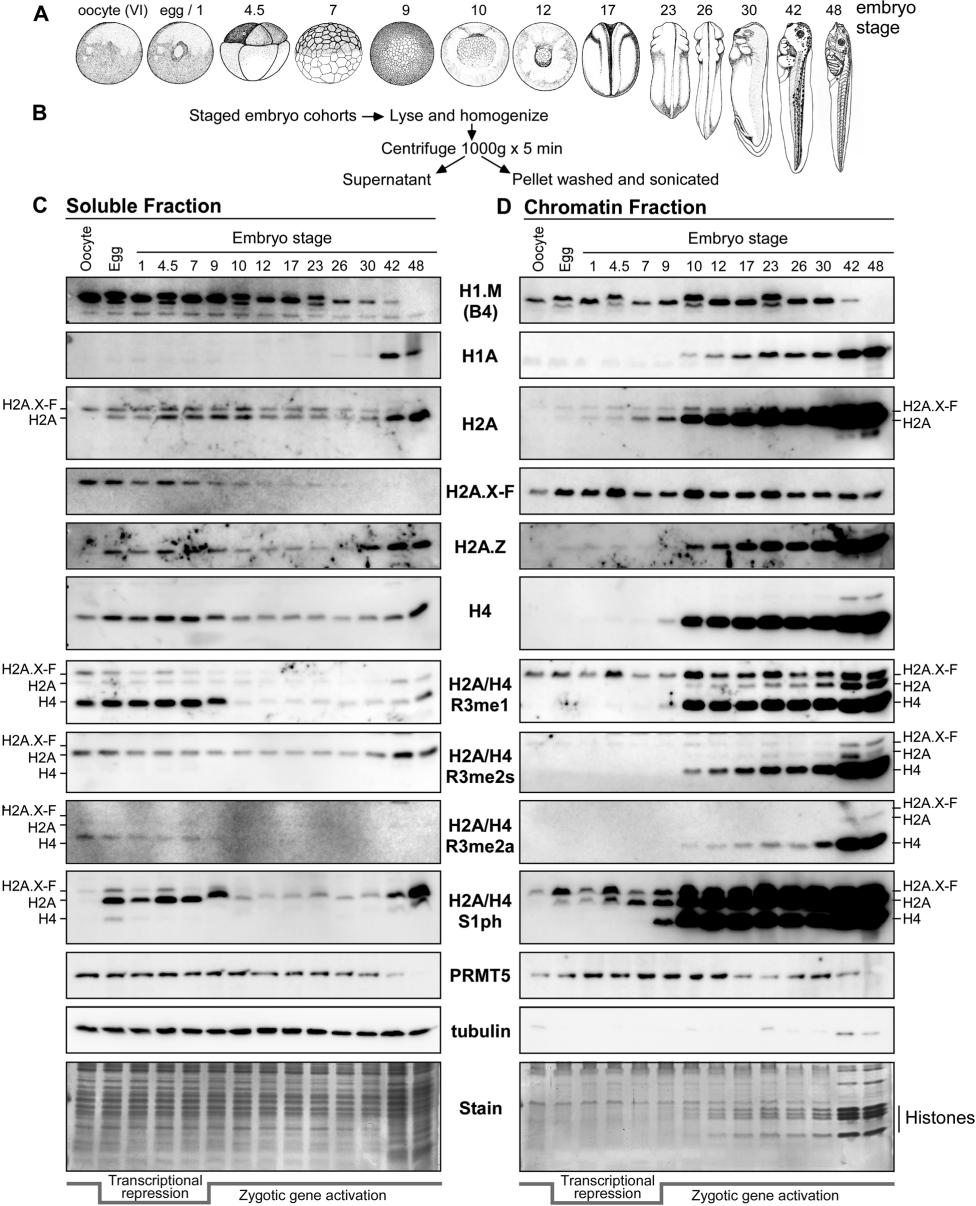
**Soluble and chromatin-bound histone isolation reveals distinct patterns of H2A and H2A.X-F modification during early development. (A)** Cartoons of the embryo stages that we collected (drawings Copyright 1994 from Normal Table of Xenopus Laevis (Daudin) by Faber et al. Reproduced by permission of Garland Science/Taylor & Francis LLC). **(B)** Embryo fractionation scheme: five embryos per stage were collected, lysed, and homogenized, centrifuged at 1,000 g and the supernatant containing soluble histones was removed. The pellet was washed in the lysis buffer and then sonicated. This material was used as the chromatin fraction. **(C)** Equivalent volume of total soluble protein from the staged embryo fractionation was immunoblotted for linker histones, core histones, and the conserved H2A/H2A.X-F/H4 modifications as shown. Total soluble protein is shown in the Coomassie stained gel at the bottom. The period of transcriptional repression post fertilization is indicated at the bottom. The migration positions of H2A.X-F, H2A, and H4 are indicated on the left. **(D)** Equivalent volume of total chromatin protein from the staged embryo fractionation was immunoblotted for linker histones, core histones, and the conserved H2A/H2A.X-F/H4 modifications as shown. Total chromatin protein is shown in the Coomassie stained gel at the bottom. The stained histone protein bands are annotated. The period of transcriptional repression post fertilization is indicated at the bottom. The migration positions of H2A.X-F, H2A, and H4 are indicated on the right.

*Xenopus* eggs and embryos have abundant stores of histones for the rapid division and is a unique tool for discrimination between pre- and post-deposition histones. Therefore, we developed a simple approach to fractionating embryos into soluble and chromatin pools to ask if there are histone loading preferences during development from the oocyte to stage 48 tadpoles (Figure 4A, B). We flash-froze small pools of staged embryos, homogenized the frozen embryos with a motorized pestle, and centrifuged the suspension at 1,000 g. The supernatant was removed as the cytosol and the pellet was washed and sonicated and used as the chromatin fraction. We confirmed the fractionation by Coomassie staining the cytosolic and chromatin fractions and by tubulin immunostaining (Figure 4C, bottom panels). Tubulin was enriched in the cytosolic fractions, as expected, while the major protein bands in the chromatin fraction were histones, as expected. We therefore used these samples to immunostain for a collection of core and linker histones as well as histone PTMs. For each gel lane we loaded equivalent volume corresponding to half an embryo.To demonstrate the utility of our fractionation approach to target developmental transitions, we first show that embryonic linker histone H1.M (B4) is abundant in cytosol until stage 9 when it had equal abundance in chromatin, and fully disappeared after stage 42 (Figure 4C, top panel). Note that H1.M appeared to have currently unexplained embryo-specific doublets. In contrast, somatic H1A was not expressed before MBT and only showed a very faint band in stage 9 chromatin. Its chromatin incorporation increased readily after zygotic gene activation and exponential cell division at stage 9.

**Figure 5:**
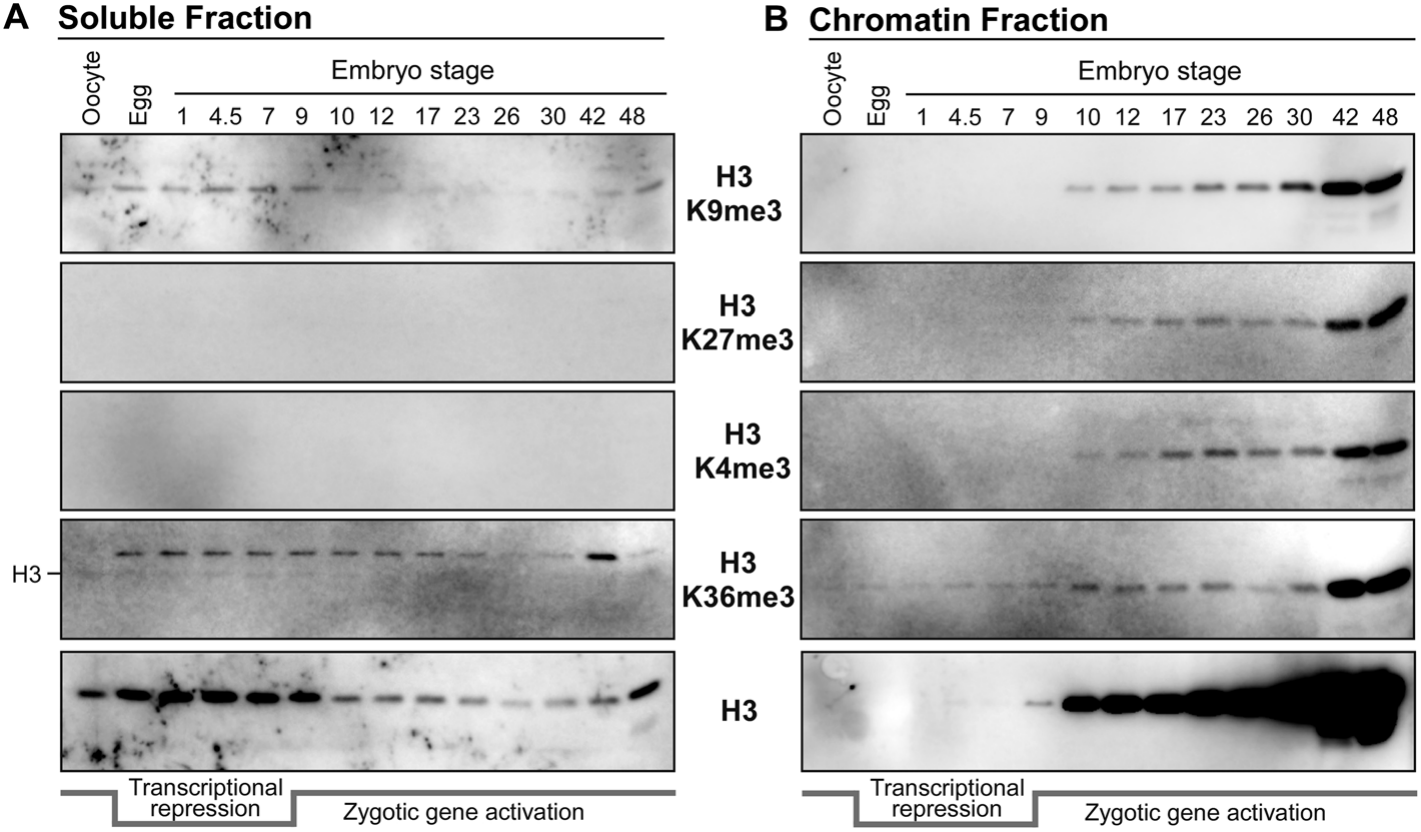
**Soluble and chromatin-bound histone isolation analysis of H3 PTMs during early development. (A)** As in Figure 3, equivalent volume of total soluble protein from the staged embryo fractionation was immunoblotted for H3, H4, and H3 K9me3, K27me3, K4me3, and K36me3. Total soluble protein is shown in the Coomassie stained gel at the bottom. The period of transcriptional repression post fertilization is indicated at the bottom. **(B)** Equivalent volume of total chromatin protein from the staged embryo fractionation was immunoblotted for H3, H4, and H3 K9me3, K27me3, K4me3, and K36me3. Total chromatin protein is shown in the Coomassie stained gel at the bottom. The stained histone protein bands are annotated.

The vast majority of histone proteins in oocytes and early embryos are stored in the cytosol in complex with chaperones such as Nucleoplasmin and N1/NASP to prevent non-specific binding before histones being incorporated in chromatin [24]. As expected, histones observed before MBT are mainly non-chromatin deposited in the soluble fraction (Figure 4C and D and Figure 5A and B). The level of stored histones sharply decreased post MBT with concomitant enrichment of core histones in chromatin. The core histones H2A, H3, and H4 had massive enrichment as the cell count increased during and after the MBT (stage 9 and on), as expected. These changes in abundance are clearly observed by our relative quantification of the histone H4 immunoblot signal (Additional file 1: Figure S4). As we had previously shown, H2A.X-F is a major histone H2A isoform in oocytes. Its abundance remains high relative to canonical H2A through the MBT and its expression decreases in the stage 9 embryo cytosol fraction. However its level in chromatin remained constant post MBT. Its occupancy in chromatin is diluted over subsequent developmental time in the absence of its new expression. Histone H2A.Z, another histone H2A variant correlated with active gene expression, was only found in chromatin post MBT, while a small soluble population was present through development.

H2A and H2A.X-F appeared to have similar relative abundances in embryo chromatin post fertilization and pre MBT (faint bands visible in stage 1, 4.5, and 7 embryo samples in the H2A blot). However, serine 1 phosphorylated H2A and H2A.X-F showed a distinct pattern in loading preference before MBT with enrichment of H2A S1ph over H2A.X-F S1ph stored in egg. In deposited chromatin, H2A.X-F S1ph is more abundant than H2A S1ph in eggs, stage 1 and stage 4.5 embryos. After stage 7, H2A S1ph relative abundance increases in the chromatin fraction, and is depleted in the cytosol. This observation suggests that H2A.X-F S1ph is preferentially loaded in chromatin.

We and others previously showed that early embryos were enriched in histone H2A and H4 R3 methylation [25-27]. To determine relative enrichments of undeposited histone H2A and H4 containing arginine methylation in the cytosol compared to that deposited in chromatin, we blotted the fractionated embryos with R3me1, R3me2s (symmetric dimethylarginine), and R3me2a (asymmetric dimethylarginine) antibodies. We had observed in the total embryo blots similar patterns as chromatin isolated from the extract produced pronuclei (Figures 1A and 3B). H2A.X-F R3me1 was present and did not change until it disappeared after stage 42. H4 R3me1 occurs at the same time, but has a gradually increasing pattern from stage 10 to stage 48. There was a relatively low abundance of H2A R3me1 in early stage embryos that increased in stage 42 and 48 embryos. Interestingly there is no H2A.X-F R3me2s in embryos while there was some H2A R3me2s present in all embryo stages, while H4 R3me2s only appeared post MBT (Figure 3B).After embryo fractionation an interesting regulation of H2A/H4 R3me deposition was revealed. Before stage 9, only H2A.X-F R3me1 was present in chromatin, with minor soluble pools (Figure 4C, D). After stage 9, H2A.X-F R3me1 remained constant in chromatin with a gradual increase of H2A R3me1 starting at stage 9 and a large quantity of H4 R3me1 appeared in chromatin. Consistent with analysis from total embryo lysates, no appreciable H2A.X-F R3me2s was detected in chromatin. In the cytosol a small amount of H2A R3me2s was detected in all stages of embryos. Most strikingly, H4 R3me2s was predominant in chromatin post MBT, while a lower signal was observed for H4 R3me2a only for much later embryo stages. The primary enzyme responsible for the H2A/H4 R3 methylation, PRMT5, was found in the cytoplasm as well as in the chromatin fraction.In comparison, histone H3 PTMs with significant roles in transcriptional regulation, such as K9me3 and K27me3 for repression and K4me3 and K36me3 for active gene transcription, were not found in soluble pools or on the chromatin until the MBT (Figure 5).

### Mass spectrometry analysis of stored and pronuclear histones H2A and H2A.X-F

To lend support to our immunoblotting, confirm PTM site localization and co-occupancies, and to assess relative PTM abundances, we performed label-free, semi-quantitative mass spectrometry analyses of heparin purified histones from *Xenopus* eggs and acid-extracted histones purified from pronuclei to compare PTMs on soluble and chromatin bound histones, respectively. For comparison with somatic cell histones we also probed *Xenopus* S3 cell histones. This approach was previously successfully used by us and others to provide orthogonal identification of histone PTMs [15,28,29]. These histone pools were further purified on a reversed-phase C8 column. H2A fractions were identified by immunoblot and Coomassie stain (Additional file 1: Figure S5).

Three separate approaches to MS sample preparation were required to optimize sample recovery, chromatography, and PTM site localization. In initial experiments, all samples were subjected to propionylation before trypsin proteolysis, resulting in peptides that are more hydrophobic [30]. This allowed for enhanced retention and better chromatographic separation using reverse-phase HPLC. Since propionylation neutralizes the positively charged lysine residues that are derivatized, it leads to a net loss of positive charge, creating peptides that are better suited to MS/MS interrogation by CAD [28,30]. Mass spectrometry analyses of H2As using trypsin revealed the relative abundance of lysine acetylations as well as arginine methylations (Table 1). Intriguingly, for the monoacetylated (not including the α-N-acetylation) species of canonical H2A in egg and pronuclei cell types, analysis of numerous MS/MS spectra showed that acetylation could be localized to both K5 and K9 (Additional file 1: Figure S6A), with the K9ac species being 10 to 100 times more abundant than the K5ac species. However, the corresponding spectra for H2A.X-F fractions from these cell types revealed that acetylation is localized to both K5 and K6 (Additional file 1: Figure S6B). Again, we estimate the K5ac species to be 10 to 100 times more abundant than the K6ac. Additionally, we found evidence for S12ph, S1ph + S12ph, R3me + K5ac, and K5ac + K6ac (Additional file 1: Figure S7). These modifications were not observed across all sample types, and when observed, their abundance was found to be less than 1%. While cleavage by trypsin is blocked at the derivatized lysine residues, cleavage at unmodified arginines yielded peptides without the first three N-terminal residues (Additional file 1: Figure S1), rendering S1ph characterization incomplete.

**Table 1:** Summary of N-terminal tail modifications observed on H2A and H2A.X-F by mass spectrometry

**A. H2A**						
			**Relative abundance on H2A**			
**Peptide**	**Modifications**	**Sites**	**Egg**	**Pronuclei**	**S2 Cells**	
1-56 (GluC)	1 Phospho	S1	•••	•••••	••	
	1 Ac	K9/K5^a^	•	••	•••	
	1 Phospho + Ac	S1 + K9^a^	---	••	---	
1-11 and 4–11 (Trypsin)	1 Me	R3	••	••		
	1 Ac	K9/K5^a^	••	••		
**B. H2A.X-F**						
			**Relative abundance on H2A.X-F**			
**Peptide**	**Modifications**	**Sites**	**Egg**		**Pronoclei**	
			H2A.X-F1	H2A.X-F2	H2A.X-F1	H2A.X-F2
1-31 (Chymotrypsin)	Unmodified (αN-Acetyl)		•••••	•••••	•••	••••
		S1	•••	•••	••••	••••
		R3	•••	•••	••	••
		R3	••	••	•	•
		K5^a^	•	••	••	••
	1 Phospho + 1 Me	S1 + R3	••	••	•••	•••
	1 Phospho + 2 Me	S1 + R3	•	•	••	••
	1 Phospho + 1 Ac	S1 + K5^a^	---	---	••	••

**A.**PTMs observed for the GluC-generated, 1–56 peptide and the tryptic peptides, 1–11 and 4–11 from canonical H2A samples. Note that in addition to the modified forms shown, unmodified and monomethylated forms were also detected (data not shown). We estimate that, taken together, unmodified (α-N-acetylated) and methylated H2A comprise approximately 80%, 20%, and 70% of the total canonical H2A in egg, pronuclei, and S3 cells, respectively. **B.**PTMs observed for the chymotrypsin generated, 1–31 peptide from H2A.X-F samples. Relative abundance values are reported as follows: −−− = not detected, • = 0.1- < 1%, •• = 1- < 10%, ••• = 10- < 25%, •••• = 25- < 50%, ••••• = ≥50%. Peptide forms that were observed at less than 0.1% relative abundance are not shown.

^a^K-Ac could often be localized to more than one location. For canonical H2A, lysine acetylation was predominantly found at K9 in egg and pronuclei samples, with some K5ac present. In S3 cells, K5ac was most abundant with some K9ac. In H2A.X-F samples across all cell types, the acetylation was primarily observed at K5 with some K6ac (K6ac approximately 10 to 100 times less abundant than K5ac). These peptides were confirmed with MS/MS and accurate mass analysis. To determine the relative abundances of each modified form of the peptide, the areas under the extracted ion chromatogram for each peptide in all of its charge states and modified forms were integrated and summed. The peak area for each modified form was divided by the total area for all unmodified and modified forms present for each peptide to obtain an estimate of the percent of each form of modified peptide.

**Figure 6:**
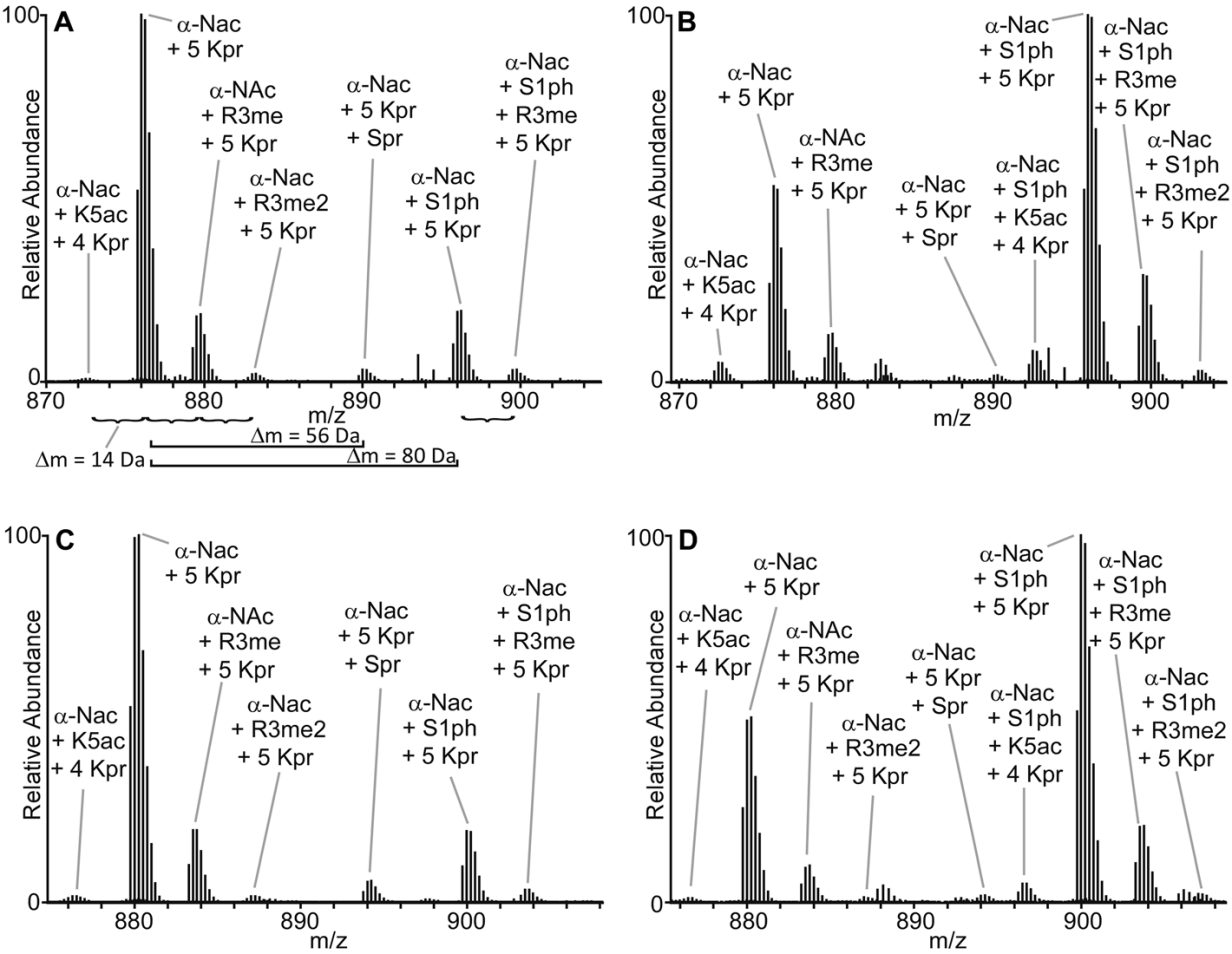
**Example high resolution MS1 scans of H2A.X-F 1–31.** Shown are high resolution MS1 scans of various PTM states seen on the [M + 4H]^+4^ ions of the 1–31 residue, chymotrypsin-generated peptides of H2A.X-F1 and -F2 from egg and pronuclei. All ions exhibiting a charge state of +4 are labeled. As an example, in panel **A**, differences of 14 Da, 56 Da, and 80 Da are noted to represent the addition of methylations/acetylations, priopionylations (Kpr and Spr), and phosphorylations, respectively. Note that H2As are 100% α-N-terminally acetylated and in the absence of acetylation, lysines are propionylated. Since the Δm for an acetylation is 42 Da while the Δm for a priopionylation is 56 Da, the K5ac form of the peptide appears at a lower mass than the α-N-acetylated (‘unmodified’) form of the peptide. Also note that serine residues can be propionylated at low levels. **(A)** H2A.X-F1 from egg. **(B)** H2A.X-F1 from pronuclei. **(C)** H2A.X-F2 from egg. **(D)** H2A.X-F2 from pronuclei.

Digestion of canonical H2A from all cell types by GluC generated the desired 1–56 peptide allowing us to characterize S1ph levels (Table 1). For H2A from S3 cells, lysine acetylation was localized to K5 and K9, with K5ac being predominant. ETD MS/MS spectra of this peptide were too complex to confidently site localize methylations. This prompted us to take a different approach with the remaining H2A.X-F histones from egg and pronuclei. These samples were propionylated and then subjected to digestion using chymotrypsin. The resulting peptides (Additional file 1: Figure S1) contained the desired S1 residue and were shorter than the GluC-generated 1–56 peptide while still containing a suitable amount of positive charge for fragmentation by ETD [31]. This simplified MS/MS spectral interpretation. Example high resolution MS1 spectra of the chymotrypsin-generated peptides are shown in Figure 6. Relative combinatorial PTM abundances are shown in Table 1.

Comparison of overall phosphorylation levels for stored egg and pronuclear H2A histones revealed that S1ph levels of all H2A isoforms are higher in pronuclei and show a three- to four-fold relative increase compared to the stored egg H2As, consistent with pronuclei immunoblots (Additional file 1: Figure S3). Over 50% of all H2A histones are S1 phosphorylated in pronuclei. Total R3 methylation is significantly higher for H2A.X-F isoforms in both eggs and early embryos compared to canonical H2A, consistent with embryo immunoblots (Figure 3B). H2A species containing lysine acetylation showed no significant increase between eggs and pronuclei, but a modest increase was observed in canonical H2A from S3 cells (Table 1).

### Analysis of S1ph and R3me1/me2s co-occupancy with known transcriptional activation and repressive PTMs

**Figure 7:**
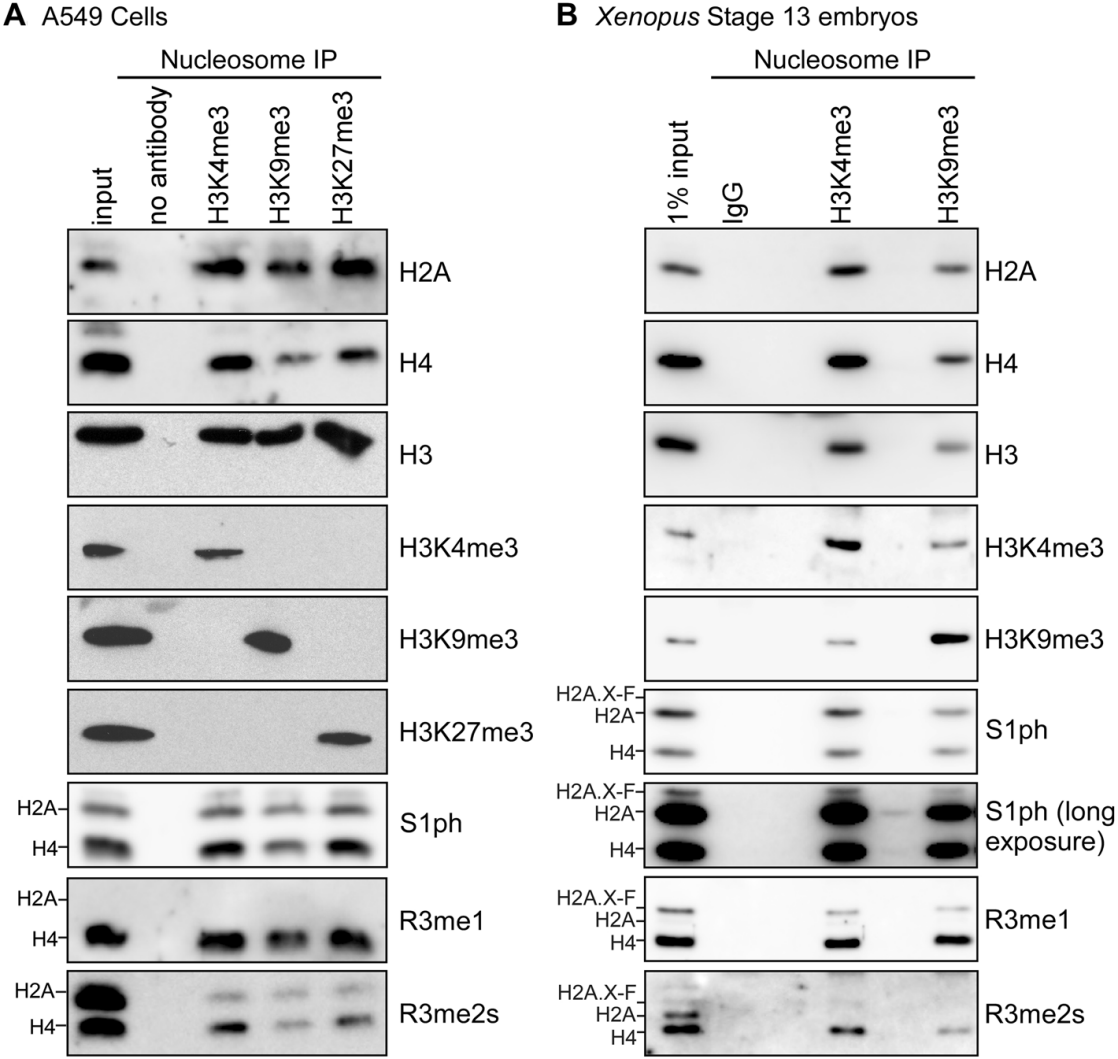
**Nucleosome immunoprecipitation demonstration of S1ph and R3me1/2 s coenrichment with active and repressive histone PTMs. (A)** Chromatin from A549 cells was digested with micrococcal nuclease and immunoprecipitated with anti-H3K4me3, H3K9me3, and H3K27me3 antibodies. Precipitated histones were blotted for H2A, H3, H4, H3K4me3, K9me3, K27me3, S1ph, R3me1, and R3me2s as indicated. **(B)** Chromatin from stage 13 Xenopus embryos was digested with micrococcal nuclease and immunoprecipitated with control IgG, anti-H3K4me3, and H3K9me3 antibodies. Precipitated histones were blotted for H2A, H3, H4, H3K4me3, K9me3, S1ph, R3me1, and R3me2s as indicated.

To determine if developmentally regulated histone H2A and H4 S1ph, R3me1 and R3me2s are correlated with transcriptional activation or repression, we performed micrococcal nuclease - chromatin immunoprecipitation experiments in cultured human cells as proof-of-principle and then in *Xenopus* embryos. First, we isolated nucleosomes from cultured A549 cells and performed chromatin immunoprecipitation with anti-H3K4me3, H3K9me3, and H3K27me3 antibodies. We normalized the precipitated histone protein concentration and blotted these histones for H2A, H3, H4, S1ph, R3me1, and R3me2s; specific immunoprecipitation was confirmed by H3K4me3, K9me3, and K27me3 immunoblots (Figure 7A). We then performed the same experiment with H3K4me3 and K9me3 on stage 13 *Xenopus* embryos (Figure 7B). Neither H2A S1ph, H2A.X-F S1ph (only visible in the long exposure) nor H4 S1ph were over- or under-enriched in any of the samples. While histone H4 monomethylarginine 3 was equally enriched in the A549 and *Xenopus* samples, H4 symmetric dimethylarginine 3 was poorly enriched in the H3K9me3 immunoprecipitations. Furthermore, histone H2A R3 methylations were substantially under-enriched in all samples.

## Discussion

**Figure 8:**
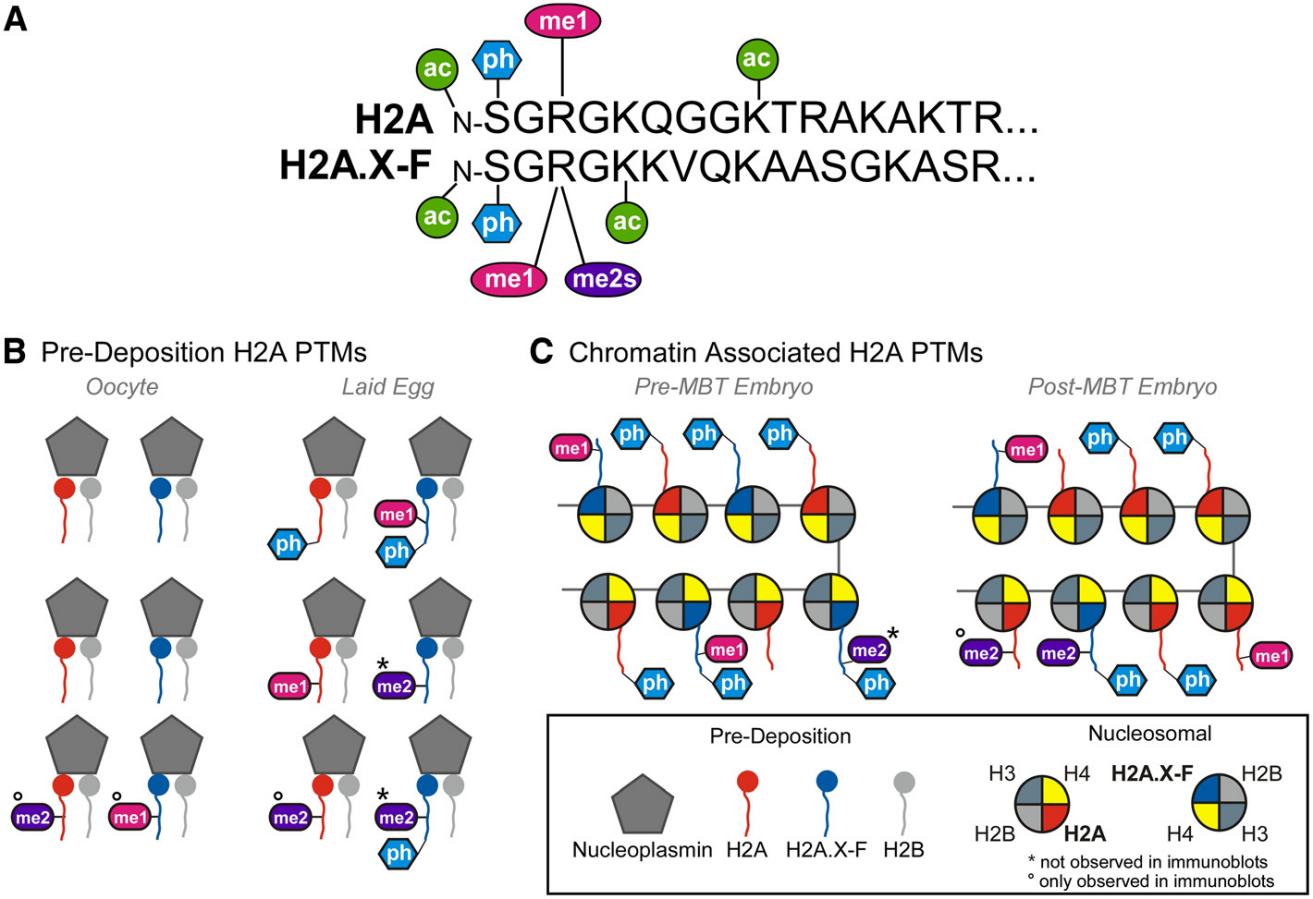
**Model of the location and timing of H2A PTMs pre and post deposition during embryogenesis. (A)** N-terminal amino acid sequence of *Xenopus laevis* canonical H2A and H2A.X-F. The modifications we observed by antibody or by mass spectrometry are illustrated: α-N-acetylated and lysine acetylation (green), Ser 1 phosphorylation (blue), Arg 3 mono-methylation (red) and dimethylation (purple). **(B)** Summary of R3 methylation (me1 and me2) and phosphorylation (ph) found on pre-deposition H2A (red) and H2A.X-F (blue) in the oocyte and laid egg soluble fractions, where they are bound to the chaperone Nucleoplasmin. * = PTMs not observed in immunoblots, ° = only observed in immunoblots. Oocyte histone PTMs were not assayed by mass spectrometry so the cartoon in the left-most column only represents immunoblot data. Co-occupancy of PTMs on a single histone tail was solely identified by mass spectrometry. **(C)** Summary of R3 methylation and phosphorylation found on chromatin associated H2A and H2A.X-F in embryos. PTMs found on pre-mid blastula transition (MBT) embryos are shown on the left, while post-MBT embryos are shown on the right. * = PTMs not observed in immunoblots, ° = PTMs only observed in immunoblots. The boxed legend references panels **B** and **C**. Co-occupancy of PTMs on a single histone tail was solely identified by mass spectrometry.

Histone H2A and H2A variant post-translational modifications have been underexplored in the literature, despite their widespread abundance across species and cell types. In this manuscript we probed the vertebrate developmental regulation of histone H2A PTMs using immunoblotting and mass spectrometry. *Xenopus laevis* oocytes, eggs, embryos, and pronuclei assembled in cell-free egg extract provided a unique opportunity to define the dynamic status of S1 phosphorylation, R3 methylation, and lysine acetylation on the canonical H2A and the early-embryo specific H2A.X-F. Our use of pronuclear assembly in egg extract and fractionation of staged embryos into soluble and chromatin fractions permitted us to unequivocally assign distinct abundances to both undeposited and chromatin bound histones using high resolution mass spectrometry. This unique characteristic of the *Xenopus laevis* model system will continue to allow dissection of pre-deposition modification of histones and their biological function.We used two complementary experimental approaches to identify these PTMs: immunoblotting, to gain temporal resolution over developmental time; and mass spectrometry, to gain precision in site identification and the ability to colocalize PTMs to the same molecule. The samples we used for mass spectrometry were primarily limited to the egg and pronuclei, which characterized the major transition between soluble, non-chromatin bound histones (egg) and those deposited into chromatin (pronuclei). The only appreciable discrepancy between the datasets is the presence of H2AR3me2s in immunoblots on developmentally staged fractions and the absence of R3me2s in the egg or pronuclear histone mass spectrometry. This difference is likely due to small sample preparation differences between these experiments, or alternatively to differences between living embryos and pronuclei assembled in cell-free egg extract. Indeed, we observed H4S1ph in pronuclear samples, but not in embryo samples until post MBT, suggesting that pronuclei histones are more equivalent to those in post-MBT embryo chromatin. The PTMs on H2A and H2A.X-F that we identified using both techniques are shown in Figure 8A. The developmental pattern of PTMs is summarized in Figure 8B and C.Our discovery of a distinct appearance of H2A/H2A.X-F S1ph from that of H4 S1ph is the first example of different deposition behavior for these two similar modifications on the same N-terminal epitope (N-SGRGK…). We showed that mono- and dimethylarginine were more abundant than previously known, strikingly on the embryo-specific H2A.X-F. Significantly, we demonstrated that undeposited, stored histones in the egg were more heavily modified on S1 and R3 (Figure 8). Our study revealed a new potential mechanism of maternal suppression of gene expression prior to zygotic gene expression, possibly mediated by H2A/H2A.X-F R3me1 and/or S1ph. As discussed below, the distinct global patterns of H2A and H4 soluble and chromatin bound histones may encode a unique signature to regulate utilization of the zygotic genome.

### Maternal regulation of zygotic development via post-translational modifications on histone variants

The mechanisms of repression of zygotic gene expression has long been enigmatic. As we previously showed, the majority of histone H2A in oocytes is the H2A.X-F variant and roughly 50% of the deposited histone H2A in embryos is H2A.X-F; here we demonstrated that this variant is heavily enriched in R3me1 and S1ph. This maternally expressed variant is diluted out of chromatin during exponential zygotic cell division when canonical H2A is expressed. We therefore favor the hypothesis that PTMs on H2A.X-F, and to a lesser extent canonical H2A, modified during oogenesis and GVBD, could be used to encode a maternal program of rapid chromatin assembly and transcriptional repression. Upon oocyte maturation to eggs, H2A and H2A.X-F contain Serine 1 phosphorylation and Arginine 3 methylation which are then deposited into embryo chromatin (Figure 8B). It is likely that that both H2A/H2A.X-F R3me and S1ph contribute to the global zygotic transcriptional repression While we do not have direct evidence to link the repression with histone PTMs, the striking correlation in the timing of H2A/H2A.X-F R3me and the zygotic transcriptional repression makes this a compelling hypothesis.

This pattern of H2A.X-F R3me1/S1ph enrichment persists through stage 9 embryos at the activation of zygotic gene expression. While we did not observe any connection between S1ph and Histone H3 K4me3, K9me3, or K27me3 in stage 13 embryos or somatic cells, future studies will examine the role of this PTM and R3me1 in pre-MBT staged embryos. Here we presented the first evidence linking histone loading preference of H2A and H2AX.F. Our unpublished evidence suggests that the disordered N-terminal tail of histone H2A may be involved in binding with histone chaperones and with chromatin remodelers (WW, DS). Future studies will test the link between H2A/H2A.X-F R3me1 and S1ph in chaperone-mediated loading preference.

The phosphorylation of histone residues can contribute to transcription activation or induction of histone acetylation [32]. Our nucleosome-ChIP studies did not show any enrichment of H2A or H4 S1ph with known activation and repressive histone PTMs in either later staged embryos or somatic human cells. This supports our hypothesis that S1ph is a component of the deposition pathway. Future studies will directly determine the distinct functions of H2A, H2A.X-F, and H4 S1 phosphorylation and R3 methylation in patterning the early embryo upon development of ChIP-Seq approaches for early-staged chromatin. Our observation in the A549 cells that H4AR3me2s, but not H2AR3me2s, appeared to be under-enriched, but not lost, in the H3K9me3 nucleosome-ChIP may be due to small differences in total histone content run in each lane; future experiments will further probe this issue. Of further interest was the under-enrichment of H2A.X-F R3me1 H2A R3me2s in the *Xenopus* H3K4me3 and H3K9me3 ChIP samples, suggesting a unique developmental role for H2A arginine methylation in the frog. If future studies demonstrate that the H3K9me3 and H2A/H4R3me2s putative silencing marks are not correlated in a genome-wide fashion, this would be consistent with distinct biological roles for these histone PTMs as suggested by genome-wide studies showing R3me2s presence at G + C enriched loci and satellite DNA [33,34].

### Functional distinction between H2A and H4 Ser1 phosphorylation

We clearly document the appearance of Ser1 phosphorylation on H2A and on H2A.X-F upon oocyte maturation and GVBD, both by specific immunostaining and by mass spectrometry. Intriguingly, we did not observe H4 S1ph before the MBT in embryos, either in the cytosol or the chromatin fractions. This modification only appears in chromatin post MBT, starting in stage 9 chromatin. This is consistent with the observations from total lysate analysis and confirmed that the phosphorylation of H4S1 happens post MBT. The total quantity of histones are similar between stage 7 and stage 9 embryos, so our observation of a massive increase of H4S1ph in stage 7 and 8 embryos is highly significant. This is the first observation of distinctly regulated timing of H2A S1ph and H4 S1ph. Previous observations in somatic cells in other organisms showed simultaneous S1 phosphorylation on H2A and H4. Our observation potentiates the discovery of novel transcriptional repression and chromatin assembly pathways in early embryos.

Chromatin assembled in pronuclei did result in H4 S1 phosphorylation in a DNA-concentration and incubation-time dependent fashion. Our previous observation of H4 S1ph in pronuclei is consistent with the results presented here [6]. The DNA:cytoplasmic ratio found at the MBT is similar to that found at the point of H4S1ph appearance in pronuclear assembly. This suggests that H4S1ph is among the many transitions that occur at the MBT in a DNA:cytoplasmic ratio-dependent fashion [23]. It also presents the intriguing possibility that distinct kinases are responsible for phosphorylation of H2A and H4 S1, or the same kinase is responsible for their phosphorylation but is activated in a DNA concentration dependent manner, despite their identical SGRGK motifs. No robust identification of kinases responsible for phosphorylation of H2A or H4 S1 have been published to date, and our attempts to identify a responsible kinase with recombinant enzymes did not show any positive activity (data not shown). Future studies will aim to identify theses kinases using conventional chromatography from egg extract.

S10ph on both H2B and H3 is correlated with meiotic chromosome condensation and disappears during meiotic divisions. H4S1ph was also linked to meiotic chromatin condensation, and it is highly enriched during sporulation in yeast and spermatogenesis in mice [10]. However, H4S1ph persists in mature spores in yeast and is eliminated post germination. In metazoans, including *Xenopus laevis*, H4S1ph is reduced late in spermatogenesis. [9]. This correlation may link H4S1ph with histone replacement by protamines [35] to produce the highly condensed nuclear structure in mature sperm. Consistently, we did not observe significant H2A or H4 S1 phosphorylation in mature sperm histone samples.

We also note the incredible density of H2A and H2A.X-F S1 phosphorylation, as we determined by semi-quantitative mass spectrometry to be roughly 15% to 20% of the total H2A in the egg and greater than 50% of the total H2A in pronuclei. The heparin chromatography that we used to enrich for soluble egg histones could under-enrich the phosphorylated histones (WW, DS, unpublished observations), perhaps leading to a conservative estimating of its abundance. These observations hint at substantial biological function, as this is a costly reaction for the egg. Such striking abundance in the egg highlights this system as an important approach for determining biological function of S1ph, which has been difficult to discern in somatic cells.

### Histone H2A arginine methylation

Arginine monomethylation had previously been dismissed as a non-biologically relevant intermediate in the production of dimethylarginine. H4R3me1 has not been correlated with any epigenetic role, nor have the equivalent arginine methylation states of H2A or H2A.X-F that are recognized by the same antibody as H4 methylation. Here, we identified monomethylarginine existence in chromatin for the first time. Symmetric di-methylation of histone H4 at arginine 3 (H4R3me2s) is linked to suppression of gene expression [36]. Symmetric dimethylation of H2A has not been functionally distinguished from that of H4.

## Conclusions

Our observations now link many important epigenetic marks to cell cycle and transcriptional transitions that occur at the mid-blastula transition and the onset of zygotic transcription. In light of our evidence presented here, the orchestration of H2A deposition in early embryos likely has strong roles in regulating pluripotency and differentiation [37]. Indeed, the incorporation of H2A.X in pronuclei is a key step in mouse embryogenesis and stem cell pluripotency [37,38]. Our data showing that early embryo stages have H2A.X-F predominantly in chromatin prior to the MBT with a gradual transition to canonical H2A is consistent with the observation in mouse embryos and stem cells. Our discovery further links histone replacement with epigenetic marks and the histone code, as the maternally expressed histones are unique in variant abundance and pre-deposition PTMs. This paper points to histone H2A and its variants as essential components of the maternal specification of zygotic development and highlights future experiments to discern their role. Further investigation of this regulation pathway will greatly promote the understanding of pluripotency and differentiation.

## Methods

### Chemicals and antibodies

Chemicals and reagents were obtained from Sigma (St. Louis, MO, USA), RPI (IL, USA) or Fisher Scientific (Pittsburgh, PA, USA). We used the following antibodies in this study: purified monoclonal Npm antibody [39], B4, H1A, Nap1 [40,41]. Anti- β-tubulin antibody (clone E7) is from the Developmental Studies Hybridoma Bank at the University of Iowa. The following antibodies were from Millipore: H2A (07–146), H2A.Z (07–594), H4 (07–108), and H2A/H4S1ph (07–179). The following antibodies were from Abcam: H3 (ab1791) and H4R3me2s (ab5823). H2A/H4R3me1 and H2A/H4R3me2s antibodies were used as described [42].

### Oocyte isolation and progesterone treatment

Frogs were handled in an ethical manner according to our animal use protocols 20121005 and 20110603 approved by the Albert Einstein College of Medicine of Yeshiva University Institutional Animal Care and Use Committee (IACUC). The following procedures were performed in strict accordance with the protocol and every effort was made to minimize suffering. *Xenopus* frogs were primed with 50 U PMSG 3 to 5 days before dissection. Frogs were anesthetized with 0.2% MS-222 (Thermo Fisher Scientific, Bridgewater, NJ, USA) pH 7.0 and sacrificed according to IACUC-approved protocols. The ovaries were immediately dissected out, and washed extensively with 1× Modified Merriam’s buffer (1× MMB: 10 mM Hepes-KOH pH 7.8, 88 mM NaCl, 3.3 mM Ca (NO_3_)_2_, 1 mM KCl, 0.41 mM CaCl_2_, 0.82 mM MgSO_4_, and 2.5 mM NaHCO_3_) to remove blood. Ovaries were cut into approximately 0.4 cm pieces and digested with liberase (Roche, Indianapolis, IN, USA) at 25°C with rotation for 2 h to release oocytes from follicular cells. Ovaries were washed with 1× MMB to remove liberase. Stage VI oocytes were isolated and incubated with 15 μM of progesterone for 0, 0.5, 1, 2, 6 h, and overnight. Ten oocytes at each time point were lysed with 50 μL extraction buffer (1X ELB, 0.5% Triton-X 100, 1× proteinase inhibitor, 1× phosphatase inhibitor, 10 mM NaButyrate, and 0.1 mg/mL cycloheximide) for 15 min, were centrifuged at 14 K rpm for 10 min, and supernatants were collected for immunoblot analysis.

### Extract preparation

*Xenopus* interphase egg extract was prepared as described [17,43]. Clarified egg extract (HSS) was prepared from low speed supernatant that was spun in an SW-55 rotor at 55,000 rpm × 45 min. The clarified middle layer was removed and respun for 30 min, and glycerol was added to 5%, aliquoted, and flash-frozen. *Xenopus* oocyte extracts were prepared from freshly dissected ovaries by disrupting the follicular layer as described above. The defolliculated oocytes were then washed extensively with 1× MMB containing 200 mm sucrose and 1 mm DTT, and the later staged oocytes settled to the bottom (the less dense stage I and II oocytes were mostly lost during the preparation). The oocytes were settled in 13 × 51-mm Beckman ultracentrifuge tubes, and excess buffer was removed. They were then spin-crushed at 35,000 rpm (150,000 × *g*) for 40 min in an SW-55 rotor. The middle layer was removed with a pipette and respun for 30 min. The middle layer was again removed, glycerol was added to 5% final, and the extract was aliquoted and flash-frozen in liquid nitrogen.

### Fractionation of embryos

Five embryos were homogenized in 100 μL of lysis buffer (10 mM Tris pH7.5, 200 mM NaCl, 5 mM MgCl_2_, 0.5% NP-40, 5 mM Na butyrate, 1× proteinase inhibitor, 1× phosphatase inhibitor, and 100 μg/mL cycloheximide) and incubated on ice for 10 min. Lysates were centrifuged in a swinging-bucket rotor for 3 min at 1,000 g to collect nuclei. Supernatants were collected as the cytoplasmic fraction, while the pellet was washed in 200 μL of lysis buffer twice and the pellet was sonicated in 40 μL of Laemlli buffer as the nuclear fraction. The cytoplasmic and nuclear fractions were diluted 10-fold prior to loading.

### Pronuclei preparation and chromatin isolation

Pronuclei were prepared by swelling 2,000 demembranated sperm chromatin per μL in 80 μL of interphase egg extract until mid-to-late S-phase, (determined by 4′,6-diamidino-2-phenylindole staining and microscopy showing the appearance of rounded, large, membranated nuclei with condensed chromatin). Samples were flash-frozen, suspended in 800 μL of ELB-CIB buffer (10 mM Hepes, pH 7.8, 250 mM sucrose, 2.5 mM MgCl_2_, 50 mM KCl, 1 mM DTT, 1 mM EDTA, 1 mM spermidine, 1 mM spermine, 0.1% Triton X-100, 10 mM sodium butyrate, 1× phosphatase inhibitors, and 1× protease inhibitors) and chromatin isolated via centrifugation at 4,000 rpm for 5 min through a 0.3 mL sucrose cushion of ELB-CIB with 0.5 M sucrose underlayered in the tube. The pellet was washed once with ELB-CIB plus 250 mM KCl. Histones from *Xenopus laevis* S3 cells were prepared as described [6].

### Immunoblotting

Lysate were run on 12% (37.5:1 acrylamide/bis) 0.75-mm-thick SDS-polyacrylamide gels, and transferred to polyvinylidene difluoride membrane (Millipore) using 1× NuPAGE transfer buffer (Invitrogen) plus 20% methanol and 0.1% SDS. Membranes were stained using Direct Blue 71 stain to ensure proper transfer; any membranes with inadequate or uneven transfer were discarded. Membranes were blocked in 2% skim milk or 3% BSA and blotted with antibodies. Secondary horseradish peroxidase-coupled antibodies were applied and then visualized using ECL Advance, with images captured using the ImageQuant LAS 4000 digital system (GE, Pittsburgh, PA, USA). Images were incrementally exposed until CCD saturation, and the exposure before saturation was used for analysis. Images were aligned with molecular weight markers by simultaneous capture of the lit membrane and subsequently cropped, and levels were adjusted for contrast in Adobe Photoshop; no exposed bands were eliminated upon adjustment.

### Sample preparations for mass spectrometry

*GluC-* C8 reverse phase-HPLC-purified fractions corresponding to canonical histone H2A from Xenopus egg, pronuclei, and S3 cultured cells (approximately 10 to 30 pmol of each protein) were reduced with dithiothreitol and alkylated with iodoacetamide as previously described [44]. Following alkylation, fractions were incubated with endoprotease GluC (Roche Applied Science) (1:20 enzyme/protein) at a concentration of 0.5 to 3 pmol/μL for 6.5 h at room temperature in 100 mM ammonium bicarbonate, pH 8. The digestions were quenched with glacial acetic acid and stored at −40°C until analysis. *Trypsin-* Purified H2A from Xenopus egg and early embryo cells (approximately 6 to 30 pmol of each protein) were reduced and alkylated [44]. Following alkylation, fractions were treated with propionylation reagent, digested with trypsin (Promega) (1:20 enzyme/protein) at a concentration of 0.5 to 2 pmol/μL for 7 h at 37°C, and immediately retreated with propionylation reagent, as previously described [30,31]. Following propionylation, samples were reconstituted in 0.1% (v/v) acetic acid and stored at −40°C until analysis. *Chymotrypsin* Purified H2A.X-F histones from Xenopus egg and early embryo cells (approximately 6 to 10 pmol of each protein) were dried and reconstituted in 100 mM ammonium bicarbonate. Samples were propionylated and then incubated with endoprotease chymotrypsin (Roche Applied Science) (1:20 enzyme/protein) at a concentration of 0.5 pmol/μL for 6.5 h at room temperature in a solution containing 1 M Guanidine HCl, 8.33 mM Tris–HCl, and 0.67 mM DTT, pH 7.8. The digestions were quenched with glacial acetic acid and stored at −40°C until analysis.

### Mass spectrometry

*GluC and trypsin:* Approximately 3 to 15 pmols of digested canonical and H2A.X-F1 & F2 peptides were pressure loaded onto 360-μm o.d. × 75-μm i.d. fused-silica micro-capillary pre-columns (Polymicro Technologies) packed with 8-cm of irregular C_18_ resin (5 to 20 μm, 120 –Å, YMC) and washed with approximately 20 column volumes of 0.1% v/v acetic acid. The precolumn was then connected to a 360-μm o.d. × 50-μm i.d. analytical column (Polymicro Technologies) packed with 6 cm of C_18_ resin (5 μm, 120 –Å, YMC) and equipped with a laser-pulled (P-2000, Sutter Instruments) electrospray emitter tip [45]. *Chymotrypsin:* Roughly 2 to 5 pmol of chymotrypsin digested H2A.X-F1 & F2 peptides were pressure-loaded onto 360-μm o.d. × 75-μm i.d. fused-silica micro-capillary pre-columns (Polymicro Technologies) packed with 5 cm of C_18_ resin (Reprosil-Pur 120 -Å C18-AQ, 5 μm diameter, Dr. Maisch GmbH) and washed with approximately 20 column volumes of 0.1% v/v acetic acid. The pre-column was then connected to a 360-μm o.d. × 50-μm i.d. analytical column (Polymicro Technologies) packed with 6 cm of C_18_ resin (Reprosil-Pur 120 -Å C18-AQ, 3 μm diameter, Dr. Maisch GmbH) and equipped with a laser-pulled (P-2000, Sutter Instruments) electrospray emitter tip [45].

All samples were gradient-eluted by nanoflow (60 nL/min) reverse-phase HPLC as previously described [28] and ionized using micro electrospray ionization [46] into a linear ion trap and Fourier transform hybrid mass spectrometer (LTQ-FTMS or LTQ-Orbitrap, Thermo Fisher Scientific). Mass spectrometers were front-end electron transfer dissociation (ETD)-enabled to allow both collisionally activated dissociation (CAD) and ETD analyses in addition to high resolution precursor mass measurements [47,48]. Both instruments were operated in a data-dependent mode in which the MS1 scan was taken from m/*z* 300–2,000 in the Orbitrap (*r* = 60,000 at m/*z* 400) or ion cyclotron resonance (ICR) (*r* = 100,000 at m/*z* 400) mass analyzer, followed by either 10 collisionally activated dissociation (CAD) MS/MS scans (prop/tryp/prop samples), or five CAD and ETD (toggled) MS/MS scan events (prop/chym) in the linear ion trap (IT) mass analyzer. For toggled analyses, MS2 parameters were set as follows: 20 to 35 ms ETD reaction time, 2E5 reagent AGC target with azulene as the electron transfer reagent, 6E4 ITMSn AGC target, CAD normalized collision energy of 35%, 3 m/*z* precursor isolation window, charge state exclusion ON for +1 precursor charge state, dynamic exclusion of 25 s with a repeat count of 2. For GluC-digested samples, mass analyses were completed with one high resolution (*r* = 100,000 at 400 m/*z*) MS1 scan followed by four low-resolution ETD MS/MS scans and two high-resolution (*r* = 60,000 at 400 m/*z* in the Orbitrap or *r* = 50,000 m/*z* in the ICR mass analyzer) ETD MS/MS scans. ETD MS2 parameters were set as follows: 10 to 20 ms ETD reaction time, 2E5 reagent AGC target, 6E4 ITMSn AGC target, 1E7 FTMSn AGC target, 3 m/*z* precursor isolation window for IT MSn, 10 m/*z* precursor isolation window for FT MSn, charge state exclusion ON for +1 and +2 precursor charge states.

### MS data analysis

High-resolution data were manually inspected using Qual Browser (Thermo Fisher Scientific) software for all masses, at abundances of 0.1% or above relative to the most abundant form, corresponding to N-terminal peptides and identified. All PTM identifications were confirmed by mass accuracy and MS/MS. Masses that agreed to within 5 ppm of the theoretical mass were further analyzed to determine their relative abundance with respect to all other modifications and combinations of modifications. Relative abundance information for each peptide species was determined by taking the area under the curve of the selected ion chromatogram for the most abundant isotope for every charge state present utilizing a 10-ppm mass window and by comparing it with the sum of the areas for every modified form of the given peptide [49]. For all species found in the high resolution analyses, CAD/ETD MS/MS data were manually interpreted for sequence validation and PTM localization.

### Chromatin assembly assay with biotin-labeled DNA fragments

Strepavidin beads (Invitrogen M280) were washed once with Dynabeads kilobase binder solution (BS) and a 1,543 bp DNA was bound at 1.43 mL beads/1 mg dsDNA and incubate with rotation at RT for 3 h. Beads were washed with 8 vol of washing solution (10 mM Tris pH 7.5, 1 mM EDTA, 2 M NaCl), and once with 8 vol of water. Each assembly reaction contained 5 uL of DNA-bound beads with 40 μL of HSS pre-incubated with energy mix and Neutravidin (10 μg/mL) at 23°C for 30 min. Samples collected from each time point were washed twice briefly with 200 μL of wash buffer (50 mM Tris pH 7.5, 10 mM EDTA, 75 mM NaCl) twice briefly with buffer containing 125 mM NaCl, and twice briefly with wash buffer containing 175 mM NaCl. Total bound samples were then run on SDS-PAGE and immunoblotted.

### Cultured cell mononucleosome immunoprecipitation

The mononucleosome immunopreciptionation was performed mostly as described [50]. Four 15 cm dishes of 80% to 90% confluent A549 cells were pelleted and resuspended in 1 mL of detergent lysis buffer (10 mM Tris, pH 7.9, 0.1% Triton X-100, 100 mM NaCl, 1 mM EDTA, 5% glycerol, 1 mM DTT, 1× protease inhibitor cocktail) at 4°C for 30 min. After centrifugation at 4,500 rpm for 10 min, the resulting nuclear pellet (approximately 60 μL) was resuspended in 180 μL of MNase buffer (50 mM Tris pH 7.4, 25 mM KCl, 12.5% glycerol, 10 mM CaCl_2_ and 4 mM MgCl_2_) with 22 U/μL micrococcal nuclease (New England Biolabs), digested at 37°C for 15 min, and stopped with 20.83 μL MNase stop buffer (200 mM EDTA, 20 mM Tris 7.9, and 0.1 mg/mL RNAse). Fifteen microliters supernatant of the reaction was used as input and another 10 μL was used to confirm digestion to mononucleosomes after protein extraction. For each immunoprecipitation, 60 μL supernatant of the MNase reaction was mixed with 80 μL HEGTw/300 buffer (20 mM Tris PH 7.9, 1 mM EDTA, 5% Glycerol, 0.1% Tween-20, 300 mM NaCl), 13 μL Protein A beads and either no antibody (negative control), or antibody against H3K4me3 (Millipore 04–745, 5 μL), H3K9me3 (Abcam ab8898, 5 μL), or H3K27me3 (Millipore 07–449, 5 μL). The reactions were rocked at 4°C for 2 h and beads washed three times in HEGTw/300 buffer. The beads were then treated with elution buffer (100 mM sodium bicarbonate, 1% SDS) at room temperature for 20 min. The eluted mono-nucleosome complexes were run on 20% SDS polyacrylamide gels for H2A, H4, H3, H2A/H4 S1ph, H3, H3K4me3, H3K9me3, and H3K27me3 detection, respectively.

### Xenopus embryo mononucleosome immunoprecipitation

Forty stage 13 embryos were suspended in 400 μL of lysis buffer (10 mM Tris pH 7.5, 200 mM NaCl, 5 mM MgCl_2_, 0.5% NP40, 5 mM Butyrate, and 1× protease inhibitors). Nuclei were pelleted via centrifugation at 1,000 g for 1 min at 4°C and washed twice with 1 mL of lysis buffer. The nuclear pellet was resuspended in 1 mL of (10 mM Tris pH 7.5, 15 mM NaCl, 5 mM MgCl_2_, 60 mM KCl, 300 mM sucrose, 1 mM DTT, 5 mM Butyrate, and 1× protease inhibitors) and centrifuged at 4,500 rpm for 5 min. The pellet was resuspended on ice gently in 100 μL of MNase digestion buffer (50 mM Tris pH7.5, 4 mM MgCl_2_, 1 mM CaCl_2_, 0.32 M Sucrose, 50 μg/mL BSA, 0.5 mM PMSF, 0.5 mM Benzamidine, 5 mM Butyrate, 1× Complete). Two microliters of the suspension was added to 100 μL of 2 M NaCl and bath sonicated for 30 s with vigorous vortexing. DNA concentration in the nuclei suspension was determined by A_260_ measurement (Nanodrop) and a final DNA suspension between 1.2 and 1.6 mg/mL was prepared by dilution with CaCl_2_ added to a final concentration of 1 mM. The suspension was equilibrated in 37°C water bath for 5 to 10 min, digested with 4 U micrococcal nuclease/70 μg DNA for 24 min, and stopped by addition of EDTA to 5 mM final and placed on ice. Debris was pelleted at maximum speed in an Eppendorf centrifuge at 4°C for 5 min and the supernatant was recovered. The extent of digestion was determined by 1.2% agarose electrophoresis. A total of 100 μl of mononucleosome solution was precipitated with 4 μg of IgG, anti-H3K4me3, anti-H3K3me3, or 4 μL of anti-S1ph serum and 20 μL of Protein A Sepharose at 4°C with rotation for 6 h. Beds were washed three times. Total bound samples were then run on SDS-PAGE and immunoblotted.

## Abbreviations

CAD: Collisionally activated dissociation; ETD: Electron transfer dissociation; GVBD: Germinal vesicle breakdown; HSS: High-speed supernatant (egg extract); K5ac: Lysine 5 acetylation; MBT: Mid-blastula transition; PTMs: Post-translational modifications; R3me1: Arginine 3 methylation; R3me2a: Arginine 3 asymmetric dimethylation; R3me2s: Arginine 3 symmetric dimethylation; S1ph: Serine 1 phosphorylation.

## Competing interests

The authors declare that they have no competing interests.

## Authors’ contributions

WW conceived of and performed experiments, interpreted results, and co-authored the manuscript. LCA and JJN performed mass spectrometry experiments and interpreted results. HC performed mononucleosome IP experiments. MJG directed and supervised the mononucleosome IP experiments. JS and DFH directed and interpreted the mass spectrometry experiments. DS conceived of and interpreted experiments and authored the manuscript. All authors read and approved the final manuscript.

## Supplementary Material

Additional file 1: Figures S1-S7 Contain data detailing the following: the H2A protease cleavage sites **(Figure S1)**; H2A and H4 S1ph antibody specificity **(Figure S2)**; egg and oocyte extract chromatin assembly **(Figure S3)**; quantification of histone H4 levels in the developmental profiles **(Figure S4)**; reversed-phase separation of histones H2A, H2A.X-F1, and H2A.X-F2 **(Figure S5)**; example CAD MS/MS spectra of acetylated H2A peptides **(Figure S6)**; example CAD MS/MS spectra of H2A.X-F2 PTM-containing peptides **(Figure S7)**. Detailed figure legends are contained in each supplemental figure page within Additional file 1. Click here for file
