# Supplementary material for: Phosphorylation and arginine methylation mark histone H2A prior to deposition during Xenopus laevis development
Source: Epigenetics Chromatin. 2014 Sep 6;7:22. doi: 10.1186/1756-8935-7-22 (PMC4191874; doi:10.1186/1756-8935-7-22)
Supplement: Additional file 1: Figures S1-S7 — Contain data detailing the following: the H2A protease cleavage sites (Figure S1); H2A and H4 S1ph antibody specificity (Figure S2); egg and oocyte extract chromatin assembly (Figure S3); quantification of histone H4 levels in the developmental profiles (Figure S4); reversed-phase separation of histones H2A, H2A.X-F1, and H2A.X-F2 (Figure S5); example CAD MS/MS spectra of acetylated H2A peptides (Figure S6); example CAD MS/MS spectra of H2A.X-F2 PTM-containing peptides (Figure S7). Detailed figure legends are contained in each supplemental figure page within Additional file 1. [file 1756-8935-7-22-S1.pdf]

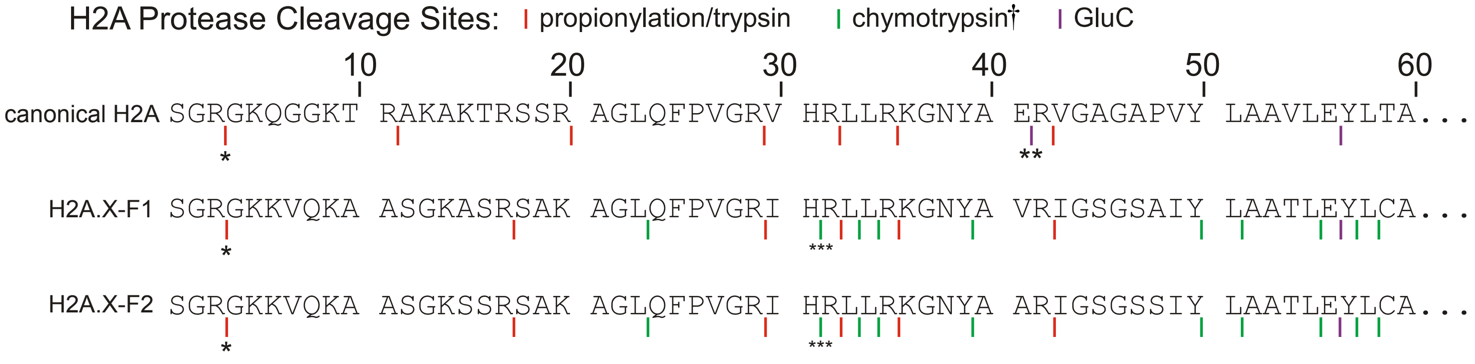

<u>*Supplemental Figure S1. H2A protease cleavage sites.*</u> Predicted digestion sites of trypsin following propionylation of lysine residues (red), chymotrypsin (green) and GluC (purple) on isoforms of H2A. *Note that most of the time in the presence of arginine methylation trypsin does not cleave. **For canonical H2A, the 1-41 GluC peptide was observed at very low abundance presumably due to a salt bridge formation between E41 and R42. ***H31 is not a predicted cleavage site, but was found to be the most abundant N-terminal peptide in all digests using chymotrypsin. †Chymotrypsin digests were not performed on canonical H2A.

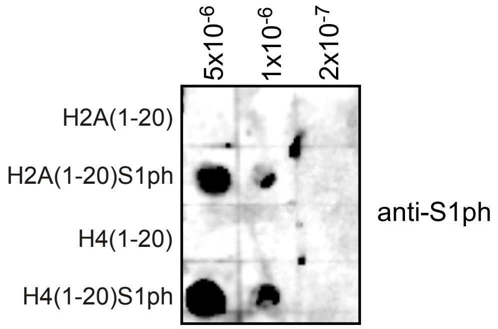

<u>*Supplemental Figure S2. S1ph antibody specificity.*</u> Dot blot demonstration of anti-S1ph equivalent specificity for H2A and H4 S1ph. Equivalent masses of H2A (1-20), H2A (1-20) S1ph, H4 (1-20), or H4 (1-20) S1ph synthesized peptides were spotted on PVDF membrane. The membrane was incubated with anti-S1ph antibody and imaged.

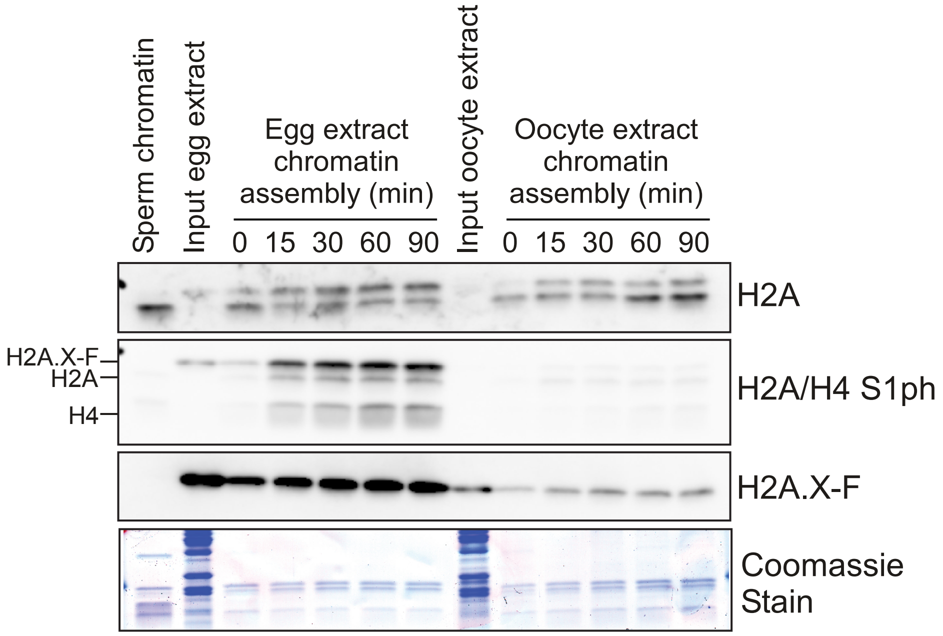

Supplemental Figure S3. Chromatin assembly in egg and oocyte extracts. Pronuclei were assembled in egg extract (left lanes) and oocyte extract (right lanes) and chromatin was isolated through a sucrose cushion at 0, 15, 30, 60, and 90 min. Isolated chromatin proteins were immunoblotted as shown. Input sperm chromatin and egg extract were also immunoblotted (left two lanes).

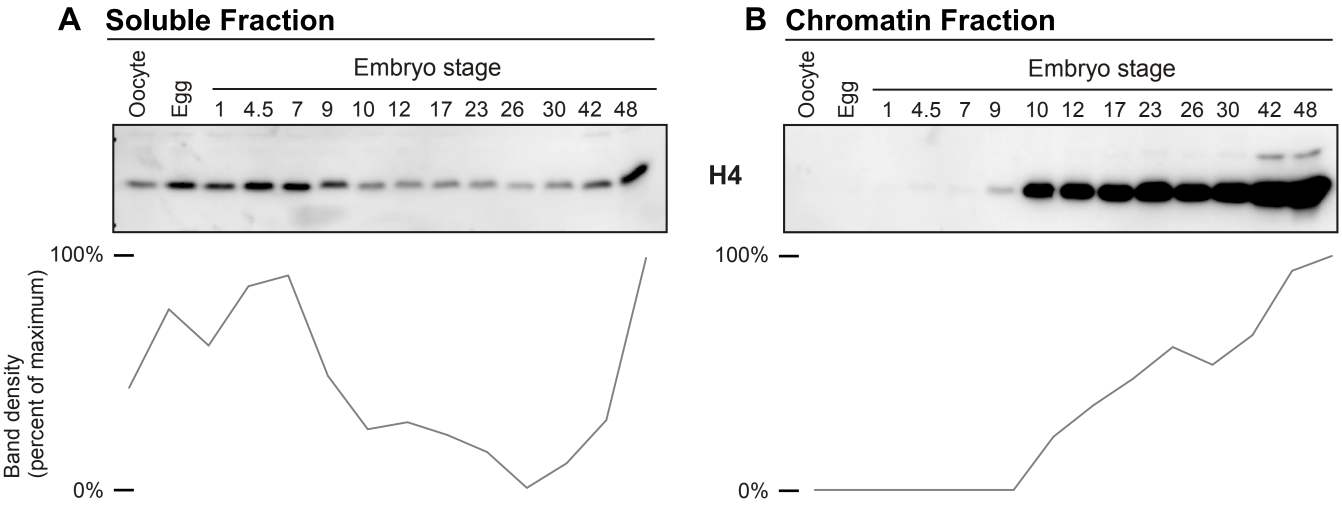

Stage	H4 (relative to max)
Oocyte	45
Egg	78
1	63
4.5	88
7	93
9	50
10	27
12	30
17	24
23	17
26	2
30	12
42	31
48	100

Stage	H4 (relative to max)
Oocyte	0
Egg	0
1	0
4.5	0
7	0
9	0
10	23
12	36
17	48
23	61
26	53
30	66
42	94
48	100

Supplemental Figure S4. Quantification of H4 immunoblot from embryos. The soluble **(A)** and chromatin **(B)** fractions blotted for histone H4 are reproduced from Figure 4. We quantitated the band density from exposures without saturation in ImageQuant TL and calculated each band's relative abundance to the densest band in each gel as a percentage. This value was plotted below the gel and corresponded to the developmental stage shown.

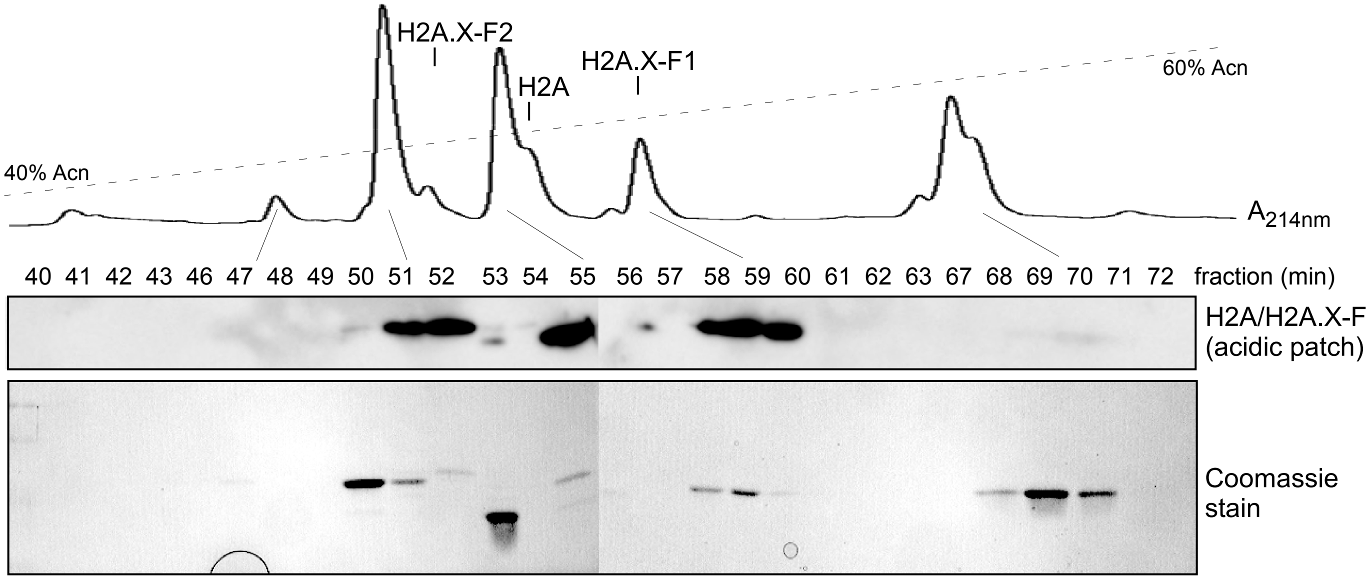

<u>*Supplemental Figure S5. Isolation and separation of H2A and H2A.X-F from eggs and pronuclei*</u>. Acid-extracted pronuclear histones were applied to a C8 reversed phase HPLC column and eluted with a gradient of acetonitrile (Acn). The UV absorbance (214 nm) chromatogram is shown at the top, with the known elution positions of H2A.X-F2, H2A, and H2A.X-F1 indicated. Fractions were lyophilized and immunoblotted for H2A and H2A.X-F (recognized by the H2A acidic patch antibody; top panel) and Coomassie stained (bottom panel).

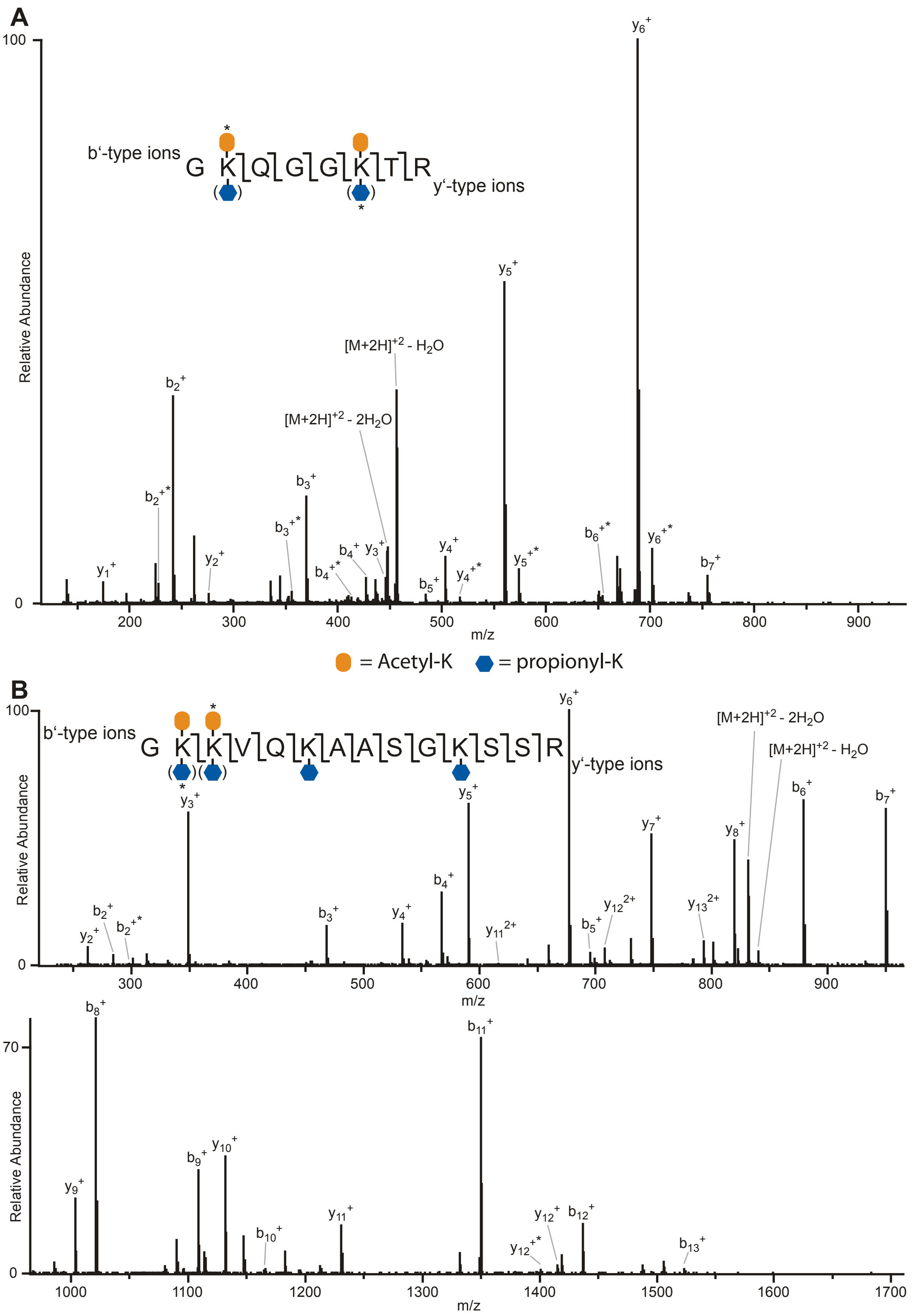

<u>*Supplemental Figure S6. CAD MS/MS Spectra of H2A peptides containing one acetylation.*</u> **(A)** A spectrum from CAD fragmentation of the canonical H2A 4-11 peptide from egg is shown. This spectrum is mixed with monoacetylation present separately on K5 and K9. In this sample, K-Ac was predominantly found at K9 with some K5ac. Fragment ions marked with an *asterisk* denote fragments that are unique to the K5ac form of the peptide. **(B)** A spectrum from CAD fragmentation of the H2A.X-F2 4-17 peptide from pronuclei is shown. Again, this spectrum is mixed with monoacetylation present separately on K5 and K6. The K5ac form is predominant. Fragment ions marked with an asterisk denote fragments that are unique to the K6ac form of the peptide. Note that in the absence of an acetyl group, lysines are propionylated. Also shown in the figure is the primary amino acid sequence each peptide, with the observed b- and y-ion indicated.

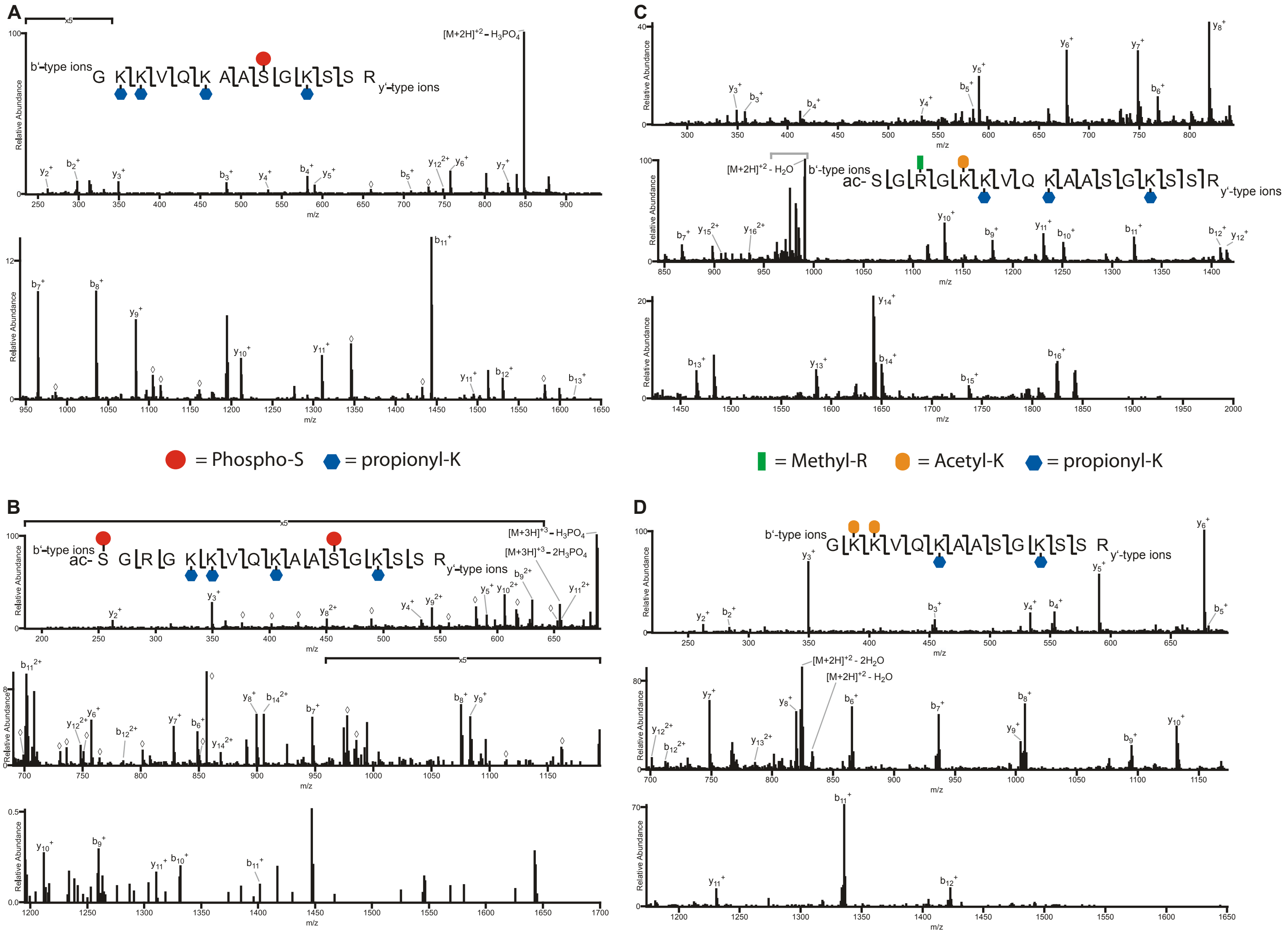

Supplemental Figure S7. Example CAD MS/MS Spectra of H2A.X-F2 peptides containing PTMs. **(A)** CAD MS/MS spectrum recorded on the $[M+2H]^{+2}$ ion (m/z 896.47) corresponding to the 4-17 residue, trypsin-generated peptide of H2A.X-F2 from pronuclei. The peptide is phosphorylated at S12. The accurate mass measurement using an LTQ-Orbitrap (m/z 896.4734) and the calculated mass (m/z 896.4719) are within 1.7 ppm. The primary amino acid sequence is shown with observed b- and y-ions indicated. Ions in the spectrum are labeled accordingly. Diamonds (◊) designate fragment ions that have undergone gas-phase ß-elimination of phosphoric acid (-98 Da from the expected fragment ion mass). **(B)** CAD MS/MS spectrum recorded on the $[M+3H]^{+3}$ ion (720.02) corresponding to the 1-17 residue, trypsin-generated peptide of H2A.X-F2 from pronuclei. The peptide is phosphorylated at both S1 and S12. The accurate mass measurement using an LTQ-Orbitrap (m/z 720. 0193) and the calculated mass (m/z 720.0188) are within 0.7 ppm. The primary amino acid sequence is shown with observed b- and y-ions indicated. Ions in the spectrum are labeled accordingly. Diamonds (◊) designate fragment ions that have undergone gas-phase ß-elimination of phosphoric acid (-98 Da from the expected fragment ion mass). **(C)** CAD MS/MS spectrum recorded on the $[M+2H]^{+2}$ ion (m/z 999.56) corresponding to the 1-17 residue, trypsin-generated peptide of H2A.X-F2 from pronuclei. The peptide is methylated at R3 and acetylated at K5. The accurate mass measurement using an LTQ-Orbitrap (m/z 999.5596) and the calculated mass (m/z 999.5582) are within 1.4 ppm. The primary amino acid sequence is shown with observed b- and y-ions indicated. Ions in the spectrum are labeled accordingly. Ions corresponding to neutral losses from the precursor are shown under the grey brackets. **(D)** CAD MS/MS spectrum recorded on the $[M+2H]^{2+}$ ion (m/z 842.47) corresponding to the 4-17 residue, trypsin-generated peptide of H2A.X-F2 from pronuclei. The peptide is diacetylated on K5 and K6. The accurate mass measurement using an LTQ-Orbitrap (m/z 842.4746) and the calculated mass (m/z 842.4731) are within 1.8 ppm. The primary amino acid sequence is shown with observed b- and y-ions indicated. Ions in the spectrum are labeled accordingly.
